# Phosphorylated tau in cerebrospinal fluid-derived extracellular vesicles in Alzheimer’s disease: a pilot study

**DOI:** 10.1038/s41598-024-75406-0

**Published:** 2024-10-25

**Authors:** Roman Sattarov, Megan Havers, Camilla Orbjörn, Erik Stomrud, Shorena Janelidze, Thomas Laurell, Niklas Mattsson-Carlgren

**Affiliations:** 1 Clinical Memory Research Unit, Department of Clinical Sciences Malmö, Lund University, Lund, Sweden; 2 Department of Biomedical Engineering, Lund University, Lund, Sweden; 3 Memory Clinic, Skåne University Hospital, Malmö, Sweden; 4 Wallenberg Center for Molecular Medicine, Lund University, Lund, Sweden; 5 Department of Neurology, Skåne University Hospital, Lund, Sweden

**Keywords:** Alzheimer’s disease, Extracellular vesicles, P-tau181, P-tau217, Biomarkers, Acoustic trapping, Alzheimer's disease, Cellular neuroscience, Assay systems

## Abstract

Alzheimer’s disease (AD) is a debilitating neurodegenerative disorder characterized by brain aggregation of β-amyloid (Aβ) peptides and phosphorylated tau (P-tau) proteins. Extracellular vesicles (EVs) can be isolated and studied for potential roles in disease. While several studies have tested plasma-derived EVs in AD, few have analyzed EVs from cerebrospinal fluid (CSF), which are potentially more closely related to brain changes. This study included 20 AD patients and 20 cognitively unimpaired (CU) participants. Using a novel EV isolation method based on acoustic trapping, we isolated and purified EVs from minimal CSF volumes. EVs were lysed and analyzed by immunoassays for P-tau217 and P-tau181. Isolation was confirmed through transmission electron microscopy and the presence of EV-specific markers (CD9, CD63, CD81, ATP1A3). Nanoparticle tracking analysis revealed a high variance in EV distribution. AD patients exhibited increased P-tau181 and decreased P-tau217 in EVs, leading to a higher EV P-tau181/P-tau217 ratio compared to CU. No significant differences in EV counts or sizes were observed between AD and CU groups. This study is the first to use acoustic trapping to isolate EVs from CSF and demonstrates differential P-tau content in AD-derived EVs, warranting further research to understand the relationship between these EV changes and brain pathology.

**Supplementary Information:**

The online version contains supplementary material available at 10.1038/s41598-024-75406-0.

## Introduction

Alzheimer’s disease (AD) is the most common neurodegenerative disorder and leads to progressive cognitive decline and memory impairment^1,2^. The neuropathological hallmarks of AD are extracellular plaques composed of β-amyloid (Aβ) peptides, and intracellular neurofibrillary tangles composed of phosphorylated tau (P-tau) proteins, which are believed to lead to neurodegeneration^3^. The development of AD is influenced by an interplay of multiple risk factors, including age, genetic predisposition, and environmental factors^4^. However, despite extensive research efforts, the exact etiology and pathogenesis of AD remain incompletely understood.

Extracellular vesicles (EVs) are emerging as a novel mode of information transfer between cells^5,6^. EVs, including exosomes and microvesicles, are small membrane-bound vesicles secreted by various cell types. EVs can be split into two main classes by their mode of biogenesis: exosomes and microvesicles. Microvesicles are vesicular structures (~ 0.1–1.0 μm) shed by outward blebbing of the plasma membrane. Meanwhile, exosomes are usually smaller EVs (∼50–150 nm diameter) that arise in the endosomal system^7^. EVs carry bioactive molecules, such as proteins, nucleic acids, and lipids, playing critical roles in intercellular communication and potentially also disease pathogenesis^8^. In the brain, EVs contain various molecules associated with neuronal function and neurotransmission, thereby contributing to the reciprocal communication between neural cells, synaptic plasticity, and neuronal activity^9^.

Due to current limitations in technology, there is little known about which proteins EVs carry. Numerous studies consistently highlight the crucial roles of EVs in AD pathogenesis^10–12^. In a mouse model of AD, it has been shown that the spread of tau occurred by the release of EVs containing protein and that depleting microglia reduced the propagation of tau^13^. That study showed that inhibiting EV release reduces tau propagation in both cell and mouse models^13^. Furthermore, the study by^14^ suggests that EV isolated from plasma contained more tau fragments than full-size tau when compared to free-floating in the extracellular medium. This is relevant to AD pathology, as fragmented tau has an increased tendency to aggregate compared to full-length tau^15^. While it is not clear how a protein such as tau can move from cell to cell in AD^16^, previous reports have suggested that this may involve EVs^17,18^.

Furthermore, advancements in the field of EVs have provided novel opportunities to unravel the underlying mechanisms of AD, including measurements of phosphorylated tau (P-tau) in EVs^17^ in human CSF, and the discovery of novel potential biomarkers associated with AD^19,20^. Studies have shown that multiple proteins such as (HSPA1A, NPEPPS, and PTGFRN) were significantly increased in AD CSF EVs^21^. Moreover, multiple synaptic proteins such as NPTX2, AMPA4, NLGN1, and NRXN2α have also been decreased in neuron-derived EVs from the plasma of AD patients^22^. Levels of cellular survival factors such as LRP6, HSF1, and REST in neural EVs isolated from plasma are lower in individuals with AD than in healthy individuals^23^. A decline in these cellular survival factors is associated with the progression of the disease^24–26^.

Furthermore, significant changes in plasma EV biomarkers of P-tau181, Aβ 1–42, and cathepsin were observed in EVs in AD^23^. EVs have also been shown to carry proteins related to the key pathologies in AD, such as P-tau^27^ and Aβ^28^. EVs may facilitate the spread of these proteins between cells, potentially propagating pathological changes throughout the brain^27,29^.In addition to proteins, lipids have also been found to be dysregulated in EVs from AD samples which can be attributed to the lipid imbalance associated with AD^30^. In this study, we focused on a novel technology to isolate EVs in cerebrospinal fluid (CSF) in human AD patients and quantified the EV content of specific P-tau variants that are of great importance in AD.

**Fig. 1:**
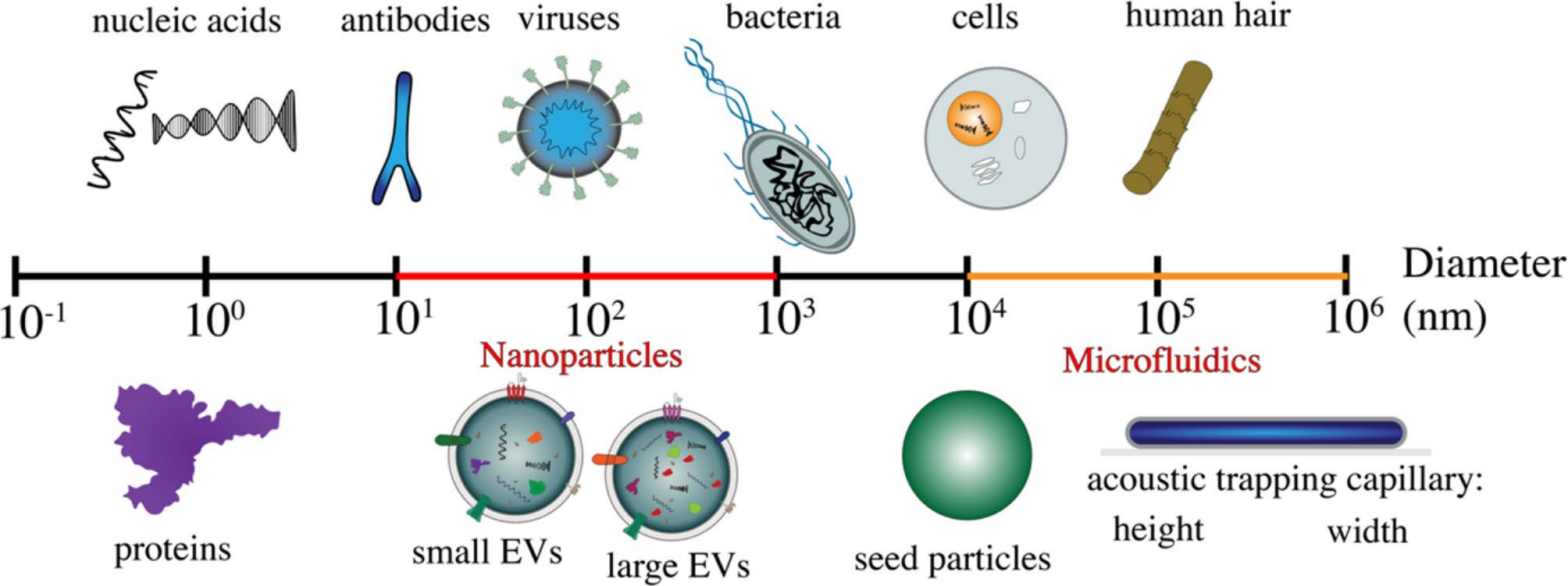
Illustration of the scale of extracellular vesicles and microfluidics compared to other biological components. This schematic provides a scale range from 1 angstrom (Å) to 1 mm (mm), mapping out the typical diameters of various biological entities in comparison to extracellular vesicles (EVs) and acoustic trapping components (seed particles and the trapping capillary) pertinent to our research.

CSF is the most informative biofluid for studies of the central nervous system (CNS)^31^. CSF is in direct contact with the brain and its content provides valuable insights into the biochemical changes occurring within the brain^32^ without the need for brain biopsies, which are inevitably ethically challenging and impractical^33^. The successful measurement of biomarkers in CSF to diagnose and monitor CNS diseases, including AD, has also raised the attention to what extent these biomarkers are EV localized or solutes in CSF. However, studies on CSF EVs present significant challenges relative to EV studies in other biofluids, including the invasive nature of CSF collection, limited CSF volumes, and the low numbers of EVs in CSF as compared to plasma^34,35^. EV content in human biofluids may vary in different studies due to differences in the isolation and detection techniques^34,36^. In this study, we investigate CSF-derived EVs from AD patients using an innovative methodology based on acoustic trapping (via the AcouTrap 2, AcouSort AB)^37^. Acoustic trapping addresses several challenges associated with traditional EV isolation methods, such as ultracentrifugation, which requires large sample volumes of CSF (0.5 ml to 18 ml)^36,38^ and is time-consuming, making it problematic when applied in clinical settings. In comparison, we demonstrate that acoustic trapping can isolate CSF EVs from 75 µl sample volumes in five minutes for acoustic trapping. Ultracentrifugation-based methods have also given inconsistent results across studies, due to variations in protocols and tools^39–41^. Furthermore, ultracentrifugation and other label-free techniques, such as size exclusion chromatography, membrane-affinity-based method or polymer-based precipitation methods, of EVs isolation vary significantly in terms of efficiency and contamination risk with lipoproteins and albumin^42^. Acoustic trapping, on the other hand, is relatively rapid, and gentle, and allows for washing, which removes solute-based proteins and results in high purity^37,43^. The size distributions of EVs in acoustically enriched samples were shifted to lower values than those obtained via ultracentrifugation^43^. This suggests that acoustic trapping not only enriches EVs but also enhances the recovery of smaller-sized EVs such as exosomes^37^. As particles become smaller, their movement becomes increasingly dominated by Brownian motion, leading to diffusion which makes separation challenging in centrifugation, filtration, and chromatography. Despite the challenge of diffusion, submicron particles have been isolated with microfluidics-based devices with dimensions approaching that of individual cells, (Fig. 1), and thus more appropriate for manipulating nanoparticles with advanced design or incorporation of acoustic, electric, and magnetic fields^39–41^.

Additionally, EVs trapped with acoustic trapping were intact and round, suggesting that the acoustic forces had negligible impact on EVs morphology^37^. Acoustically enriched EVs were functionally intact and exhibited pro-inflammatory effects^44^. The study presented herein demonstrates for the first time the enrichment of EVs from minute CSF volumes, and the simultaneous removal of free proteins, employing acoustic trapping. We investigated the morphological properties of EVs (including, shape, size distribution, and presence of tetraspanins). Furthermore, we aimed to isolate and analyze the cargo contained in these vesicles. This could allow for the isolation of biomarkers and provide possibilities for monitoring the pathological processes of AD and the biomolecular cargo of isolated EVs were analyzed, monitoring biomarkers related to pathological processes of AD. We measured levels of biomarkers within CSF-derived EVs, with a particular focus on key P-tau variants, namely P-tau181 and P-tau 217, previously measured in CSF and blood plasma for diagnosis of AD^45,46^, and elevated from the early stages of the disease^47,48^.

## Materials and methods

### Study participants and CSF sampling

For preliminary studies to optimize the acoustic trapping for CSF, we used de-identified CSF samples from 10 AD patients and 10 controls. The AD patients were selected based on a positive CSF Aβ42/Aβ40 ratio (< 0.089) and an MMSE score below 25 (mean 17.5). Samples were de-identified before analysis and provided by obtained from the Memory Clinic at Skåne University Hospital, Malmö. For the main experiments, we used CSF samples from cognitive unimpaired (CU) subjects (*n* = 20) and AD patients (*n* = 20), also obtained from the Memory Clinic at Skåne University Hospital, Malmö. CSF samples were collected by lumbar puncture using a sterile needle and transferred into sterile polypropylene tubes. Care was taken to avoid any contamination during the collection process. CSF concentrations of the established AD markers Aβ42/Aβ40 and P-tau181 were measured with Meso Scale Discovery (MSD). All AD patients had positive levels of Aβ42/Aβ40 and P-tau181, while all CU individuals had negative levels of these biomarkers. Cut-points for P-tau181 (> 50 ng/l) and Aβ42/Aβ40 ratio (< 0.089) have been defined previously^12^.

### EVs isolation from CSF using Acoustic Trapping

**Fig. 2:**
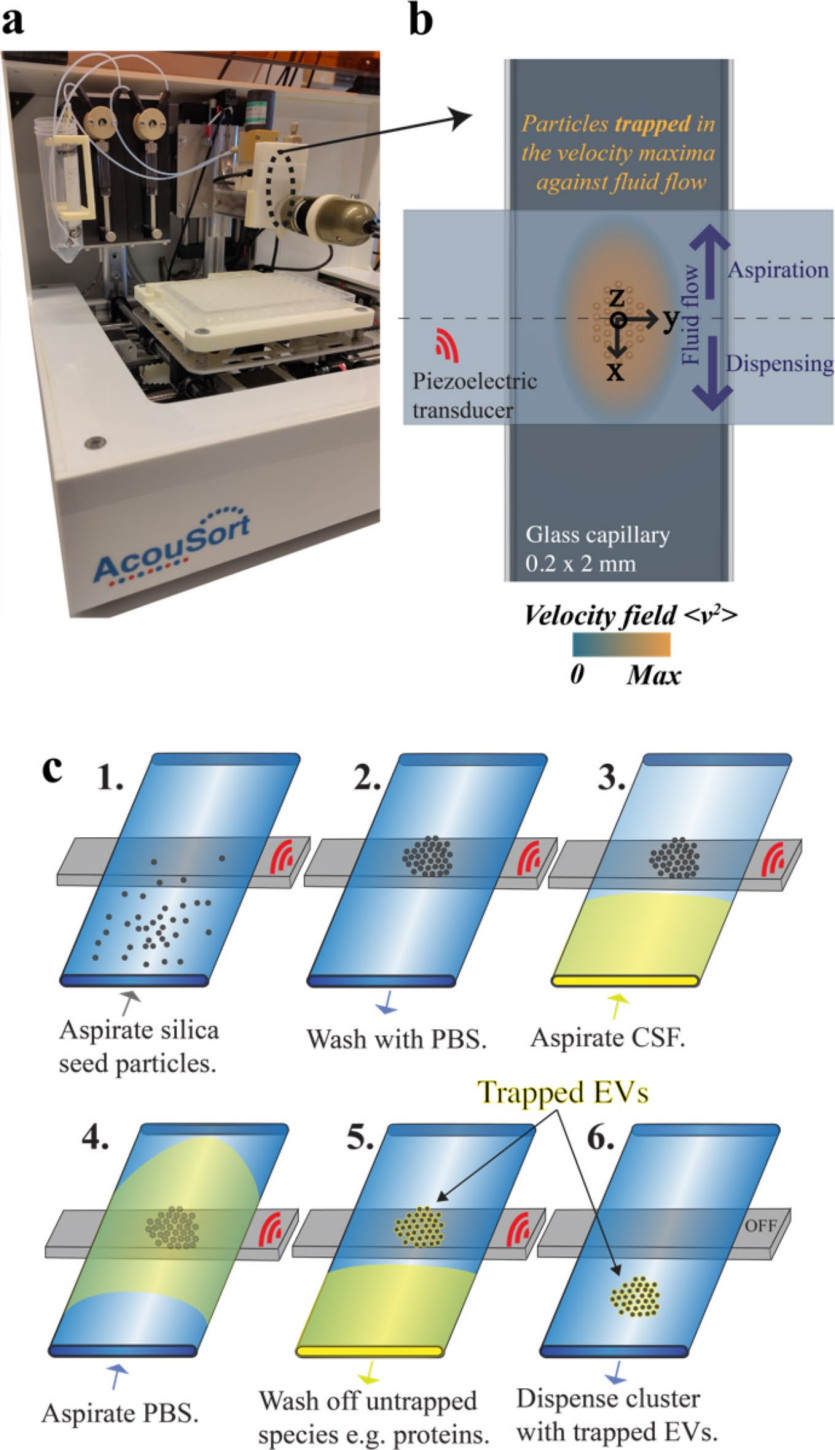
Illustration of the acoustic trapping process for extracellular vesicles (EVs). (**a**) Image of the setup (AcouTrap 2, AcouSort AB) under CC BY 4.0 (**b**) Schematic of the acoustic trapping capillary, with a rectangular cross-section of 2 mm in width and 200 μm in height. The acoustic field is generated through a piezoelectric transducer operating at 4 MHz, 10 Peak-to-peak voltage which produces a pressure node in the center of the capillary above the transducer. The generated acoustic field orchestrates particle alignment in the z direction via the pressure field gradient and the velocity field gradient (shaded in orange) retains the particles against the fluid flow in the x direction. (**c**) The depicted protocol outlines the steps for isolating EV using silica seed particles. Initially, the ultrasound is activated with frequency tracking to sustain the resonant frequency for the duration of the trapping procedure. The following steps are detailed: (**c1**) Stirring of silica seed particles (10 μm diameter) suspended in PBS for 60 s using a magnetic mixer, followed by the aspiration of 75 µl at a flow rate of 50 µl/min. (**c2**) Formation of a trapped particle cluster above the transducer by washing with 100 µl of PBS at 100 µl/min. (**c3**) Aspiration of CSF at 30 µl/min. (**c4**) Subsequent aspiration of 25 µl of PBS at the same flow rate. (**c5**) Non-trapped entities were removed by dispensing 200 µl of PBS at 100 µl/min. (**c6**) Release of the trapped EVs along with the seed particle cluster by deactivating the ultrasound and dispensing 75 µl. (inspired - Adapted from^51^ with permission from the journal Physical Review Applied.

Acoustic trapping was performed to isolate EV from the CSF samples. The setup (AcouTrap 2, AcouSort AB) consisted of an acoustic trapping device as described in^12,37^, pictured in Fig. 2a. Briefly, a glass capillary with a rectangular cross-section of 2 mm by 0.2 mm was coupled to a localized piezoelectric transducer (Fig. 2a**)**, actuated with 4 MHz frequency ultrasound at 10 V*peak-to-peak* established a local acoustic standing wave field. Micro-particles entering this field are levitated in the pressure node by acoustic radiation forces dependent on size and acoustic contrast with the medium and trapped by acoustic retention forces driven by the velocity field gradient if they have a higher density than the medium. Sub-micron particles can be trapped into a preloaded cluster of larger seed particles, for example, polystyrene 10 μm particles have been used to create a trapping site for enriching extracellular vesicles from plasma and washing away soluble components^37^. Silica seed particles have been recently shown the ability to trap nanoparticles (at concentrations < 10^*11*^ particles/mL) more efficiently than polystyrene seed particles^49^. Silica seed particles have also demonstrated higher retention force against fluid flow and thus enable higher throughput when compared to polystyrene seed particles. Acoustic trapping of EVs from CSF has not been previously published, but it is easier to handle in the AcouTrap than plasma due to the lower viscosity and concentration of non-vesicular components. All CSF samples were centrifuged at 100 g for 10 min prior to trapping to remove any large particles. Acoustic trapping protocol was performed as illustrated in (Fig. 2b). The 10 μm silica seed particles were pre-loaded into a microfluidic channel creating a stable cluster followed by 75 µl aspiration of the CSF. After the sample, phosphate-buffered saline (PBS) was aspirated to bring the CSF past the trapping region. Washing was then performed with PBS, allowing soluble compounds to be dispensed as waste and increasing the purity of the enriched EVs. The trapped EVs were collected for further analysis by turning off the ultrasound and dispensed in 75 µl into a 96-well plate below the tubing tip. Isolated samples were transferred to low-binding tubes, placed on dry ice, and then frozen at -80 °C until further use.

### Nanoparticle tracking analysis

**Fig. 3:**
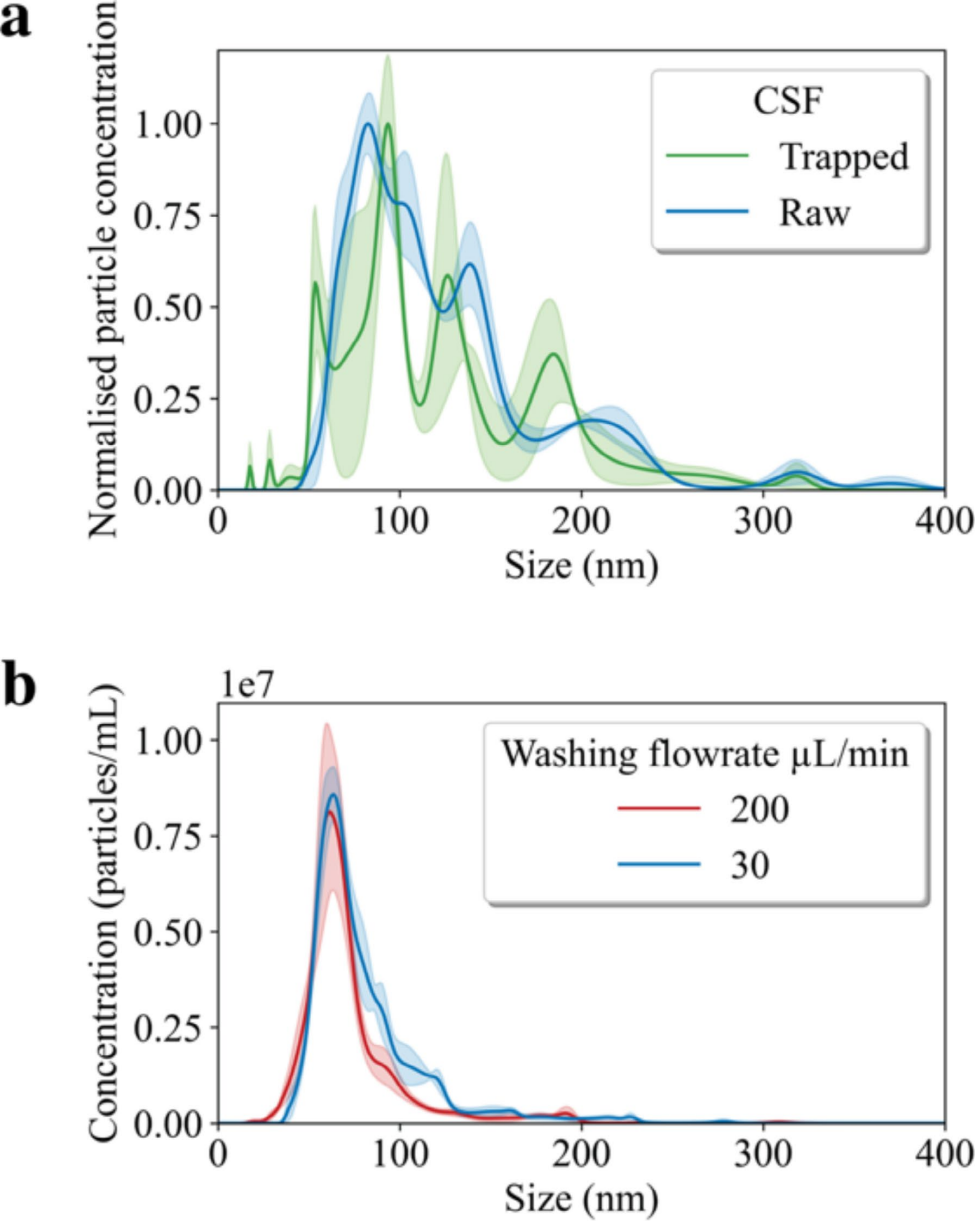
Comparative nanoparticle tracking analysis (NTA) of CSF-derived particles, extracellular vesicles (EVs). Panel **(a)** Results of NTA performed on raw-CSF (blue) and trapped (green)of a cognitively unimpaired (CU) patient. The CSF was subsequently diluted to a total volume of 1 ml in preparation for NTA. The mean particle size is 127 nm and the mode is 83 nm. Size distribution of trapped EVs from CSF. The results, aggregated from five separate captures, show that the trapped samples have a mean particle size of 145 nm mode 93 nm. The graphical profiles display an NTA analysis of EVs based on three 90-second videos capturing the trapped EVs. (**b**) Effects of different wash flow rates on EV yield using NTA. The figure shows the NTA of EVs isolated from the CSF of a healthy control subject. The EVs were trapped using two distinct washing flow rates, set to 200 µl/min or 30 µl/min, to evaluate the impact of flow rate on EV capture efficiency. In each condition, the trapping procedure was performed in triplicate, and the resultant EVs from these three runs were combined. The pooled samples were then diluted to a final volume of 1 ml to facilitate NTA measurement of the concentration of particles and different wash flow rates.

To characterize the size distributions and concentrations of nanoparticles in the CSF samples, Nanoparticle Tracking Analysis **(**NTA) was performed, using a NanoSight LM10 instrument (Malvern Analytical). NTA uses light diffraction patterns to measure the size and the concentration of EVs. For each patient (*N* = 40), the dispensed trapped sample was diluted to 1 ml with particle-free PBS to achieve sufficient volume for the measurement. The samples were then loaded into the NTA instrument, and the Brownian motion of nanoparticle diffraction patterns was tracked. The instrument software provided the size distribution and concentration information based on the recorded data (Fig. 3**)**. To generate statistical data, five 90-s videos were recorded and analyzed using NTA 3.4 Build 3.4.4 software (camera level: 15; detection threshold: 4).

### Transmission electron microscopy

CSF and trapped EVs samples derived from one AD and one control patient (selected based on high concentration of EVs according to NTA) were analyzed by Transmission Electron Microscopy **(**TEM). Copper TEM grids (400 mesh) were pre-treated with piliform, carbon coated, glow discharged. Trapped samples were vortexed and diluted with 4% PFA in a 1:1 ratio and incubated for 10 min at room temperature (RT) to fix the EVs. Then 10 µl of sample was deposited on each grid and incubated at RT for 20 min without drying out. The samples were then labeled, fixed and stained by floating on top of droplets sequentially as described. Grids were then incubated in PBS for 5 min. Followed by blocking with bovine serum albumin (1% BSA in PBS) and washing in PBS. Samples were subsequently incubated with chosen primary antibodies, diluted 1:30 in PBS-1%, for 1 h at RT. We studied the tetraspanins CD9, CD63, and CD81, which are generally considered classical EV markers^50^. Typically, small-sized EVs are CD9 positive, medium-sized EVs are CD81 positive, and larger EVs are positive for both CD81 and CD9^51^. Primary antibodies used include: CD81 Monoclonal Antibody (M38) (Catalogue # 10630D, Thermofisher), CD9 Recombinant Rabbit Monoclonal Antibody (SA35-08) (Catalog # MA5-31980, Thermofisher), CD63 Monoclonal Antibody (Ts63) (Catalog # 10628D Thermofisher), ATP1A3 Monoclonal Antibody (XVIF9-G10) (Catalog # MA3-915 Thermofisher) and GLT-1 Recombinant Rabbit Monoclonal Antibody (9H9L17) (Catalog# 701988 Thermofisher). Grids were then washed three times in PBS for 5 min each time. Followed by 40 min of incubation using appropriate secondary antibodies diluted in PBS-1% (15 nm gold colloidal goat anti-mouse and/or 10 nm gold colloidal goat anti-rabbit). The grids were washed again with PBS three times for 5 min. Samples were then placed in glutaraldehyde (1% in PBS) for 5 min at RT and then washed in distilled water for 5 min. Finally, the grids were incubated for 5 min in 1% uranyl acetate. Samples were then left to dry before sample grids were observed under a transmission electron microscope (FEI Tecnai Biotwin 120 kV).

### AD biomarkers

P-tau181 and P-tau217 were analyzed using immunoassays on the MSD platform (Meso Scale Discovery, Rockville, MD, USA) developed by Lilly Research laboratories^52^. Biotinylated (Thermo Scientific) IBA493 (Lilly) and IBA406 (Lilly) were used as capture antibodies in the P-tau217 and P-tau181 assay, respectively. SULFO-TAG (Meso Scale Discovery) conjugated 4G10-E2 antibody (Lilly) was used as the detector. The assays were calibrated with synthetic p-tau217 and ptau181 peptides^52^. The samples were thawed on wet ice. To perform the assays, MSD small-spot streptavidin-coated plates (Meso Scale Discovery) were blocked for 1 h at RT with 200 µl of 3% BSA in DPBS with 650 rpm mixing on a plate shaker. The plates were then washed three times with 200 µl of wash buffer (PBS + 0.05% Tween 20) and 25 µl of biotinylated capture antibody (either IBA 406for P-tau181 at 1 µg/ml or IBA493 for P-tau217 at 0.1 µg/ml) were added to the wells and incubated for 1 h at RT with 650 rpm shaking on a plate shaker. The plates were again washed three times with 200 µl of wash buffer. 50 µl of diluted calibrator or sample, either non-lysed control or treated sample with tween 0.5%, was added to each well and incubated for 2 h at RT with 650 rpm shaking on a plate shaker. The plates were then washed three times with 200 µl of wash buffer and 25 µl of SULFO-TAG-4G10-E2 detection antibody was added at 0.02 µg/ml to the plates and incubated for 1 h at RT with 650 rpm shaking on a plate shaker. The plates were washed a final time with 200 µl of wash buffer. Finally,150 µl of 2x MSD Read Buffer T with Surfactant (Meso Scale Discovery) was added to each plate and read on the MSD SQ120 within 10 min of read buffer addition. Samples were measured with two technical replicates and the mean of duplicates was used in statistical analysis. Levels of CSF P-tau181 were measured out prior to that (Cat. # 231654, Fujirebio Diagnostics, US, Malvern, PA) using the Lumipulse G1200 automated immunoassay platform according to the method described^53^.

### Statistical analysis

Statistical analyses were performed with the R software (version 2023.06.1). Data was checked for normal distribution using the Shapiro-Wilk test and Q-Q plot. Groups (e.g., AD versus CU) were compared with the Mann-Whitney U test (Wilcoxon rank-sum test). Scatterplots depicting the relationship between independent variables (e.g., age, sex) and dependent variables (e.g., P-tau levels) were supplemented with linear regression lines. The regression lines illustrate the predicted values based on the least squares method. Shaded areas around the regression lines represent 95% confidence intervals. Correlations between continuous data (e.g., EVs P-tau levels versus age) were tested with Spearman’s rank correlation. Biomarker levels were expressed as mean ± standard deviation (SD). A p-value threshold of 0.05 was used to denote statistical significance.

### Ethics

All procedures were conducted in accordance with relevant ethical guidelines and approved by the ethical review authority, Regional Ethical Review Board Lund (#2013/494). Informed consent was obtained from all study participants.

## Results

The main study included 20 AD patients and 20 CU individuals (Table 1). The AD group had, by definition, lower CSF Aβ42/Aβ40 and higher CSF P-tau181 and also higher P-tau217 levels compared to the CU group.

**Table 1:** Summary demographic, biological, and clinical profiles of CSF donors. Summary list of the various characteristics of individuals with Alzheimer’s disease (AD) and cognitive unimpaired (CU). Values are mean with standard deviation (SD) for each group.

Diagnosis	AD	CU
N	20	20
MMSE	17.5	28.9
Sex (male/female)	7/13	8/12
Age at sampling (years), mean ± SD	73±8	60±12
CSF Aβ42/Aβ40, mean ± SD	0.4±0.1	1.1±0.1
CSF P-tau181 (ng/l), mean ± SD	99±13	32±5
CSF P-tau217 (ng/l), mean ± SD	64±22	4±1
Trapped P-tau181 (ng/l), mean ± SD	0.4±0.2	0.2±0.05
Trapped P-tau217 (ng/l), mean ± SD	0.6±0.4	1.3±0.7
Ratio P-tau181/217 ± SD	1.7±1.9	0.22±0.1
Number of EVs trapped, mean ± SD	(5.0±0.3) x10^8^	(5.6±0.2) x10^8^
Mean diameter (nm) mean ± SD	144±57	144±59
Mode of diameter (nm) mean ± SD	121±10	116±16

### NTA of CSF and EVs

Using NTA, we quantified the particle size distribution and concentration in unprocessed CSF samples (raw-CSF) from AD (*N* = 1) and control samples (*N* = 1). Figure 3a shows the variation in particle size distribution and concentration between raw CSF samples and isolated EVs. Trapped EVs had a mean size of 62.5 nm, compared to 125.2 nm in raw CSF samples, indicating our isolation technique preferentially traps smaller particles. Additionally, Fig. 3b shows that varying the acoustic trapping flow rates did not affect the quantity of EVs trapped. During the isolation process, silica seed particles were utilized to capture EVs from CSF. The efficiency of EV trapping was assessed under different washing flow rates. Our results indicated that the trapping efficiency was consistent when the washing flow rate was increased from 30 µl/min to 200 µl/min, with no significant differences observed in the yield of EVs under these conditions. Note that the total concentration of particles in the samples measured were within the recommended range of 10^7^-10^9^ particles/ml.

### EVs size distribution

**Fig. 4:**
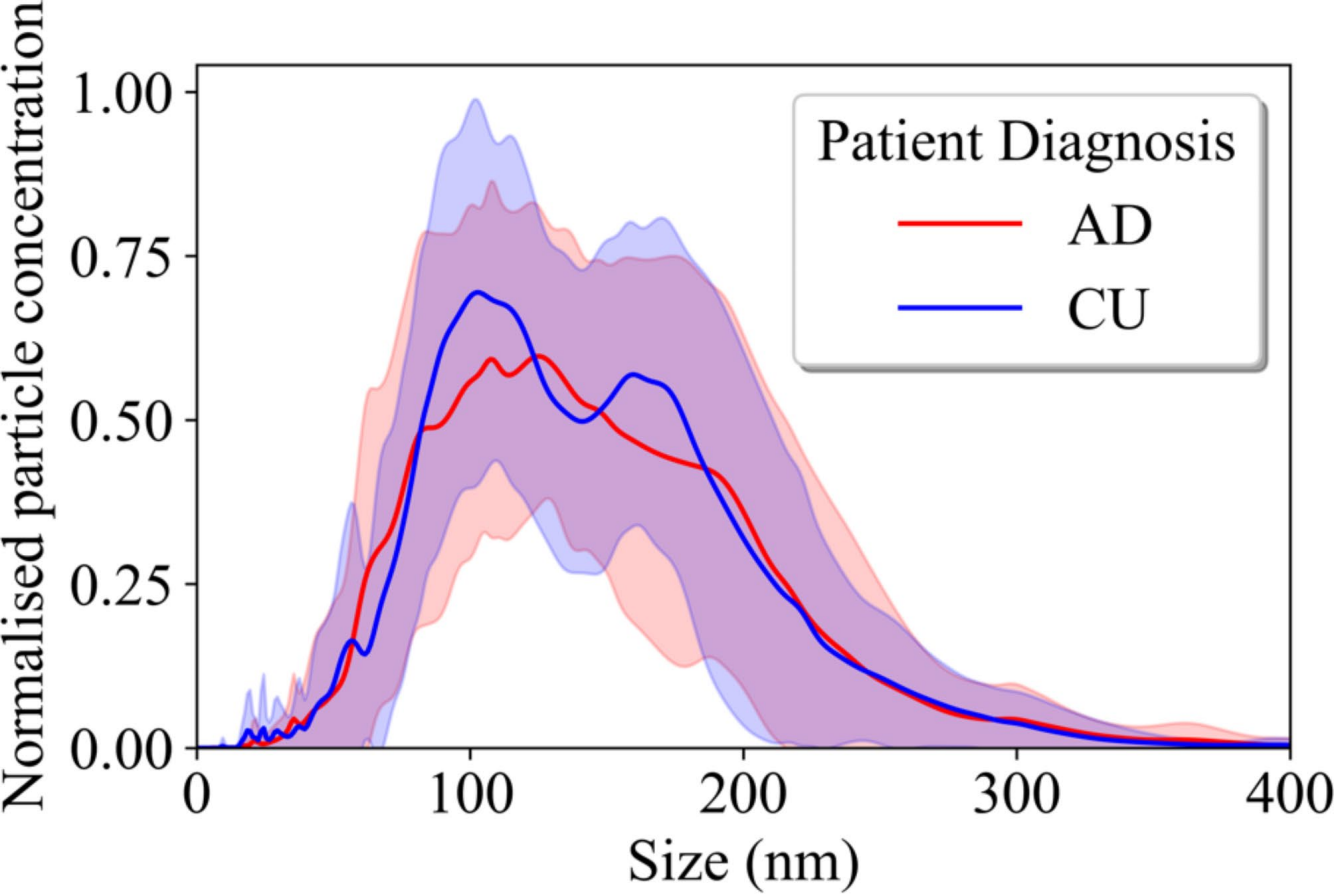
Nanoparticle tracking analysis (NTA) distribution profiles of trapped EVs from CSF in Alzheimer’s disease (AD) and cognitive unimpaired (CU). The NTA profiles represent EVs isolated from the CSF of AD patients (*N* = 20) and CU (*N* = 20). The EVs were aspirated at a volume of 75 µl with a flow rate of 200 µl. The analysis revealed a mean particle size of 144 nm across the samples AD and CU groups.

EVs from (AD, *N* = 20 and CU, *N* = 20) isolated from CSF were analyzed using NTA. As depicted in Fig. 4, variability was noted in the size of EV particles among participants. For the AD group, the mean diameter of EVs was 144.5 nm with a mean standard deviation of 57.2 nm, and the most common size (mode) was 121.0 nm. The CU group was similar, with a mean diameter of 144.0 nm, a mean standard deviation of 59.1 nm, and a mode of 116.5 nm. Our Wilcoxon test analysis indicated no statistically significant differences (p-value = 0.789) in the quantity of EVs between the AD and CU groups. Furthermore, we observed no significant variations attributable to sex or age within our study population. Additionally, there was no correlation found between the mean and mode sizes of EVs and the levels of P-tau in trapped EVs. **Supplementary Fig. 1** shows the ratio between EV concentration and mean and mode. There was a slight increase of mean in the AD group but no statistically significant difference between the groups for either mean or highest peak (mode).

### TEM immunogold labeling of EVs isolated from CSF

**Fig. 5:**
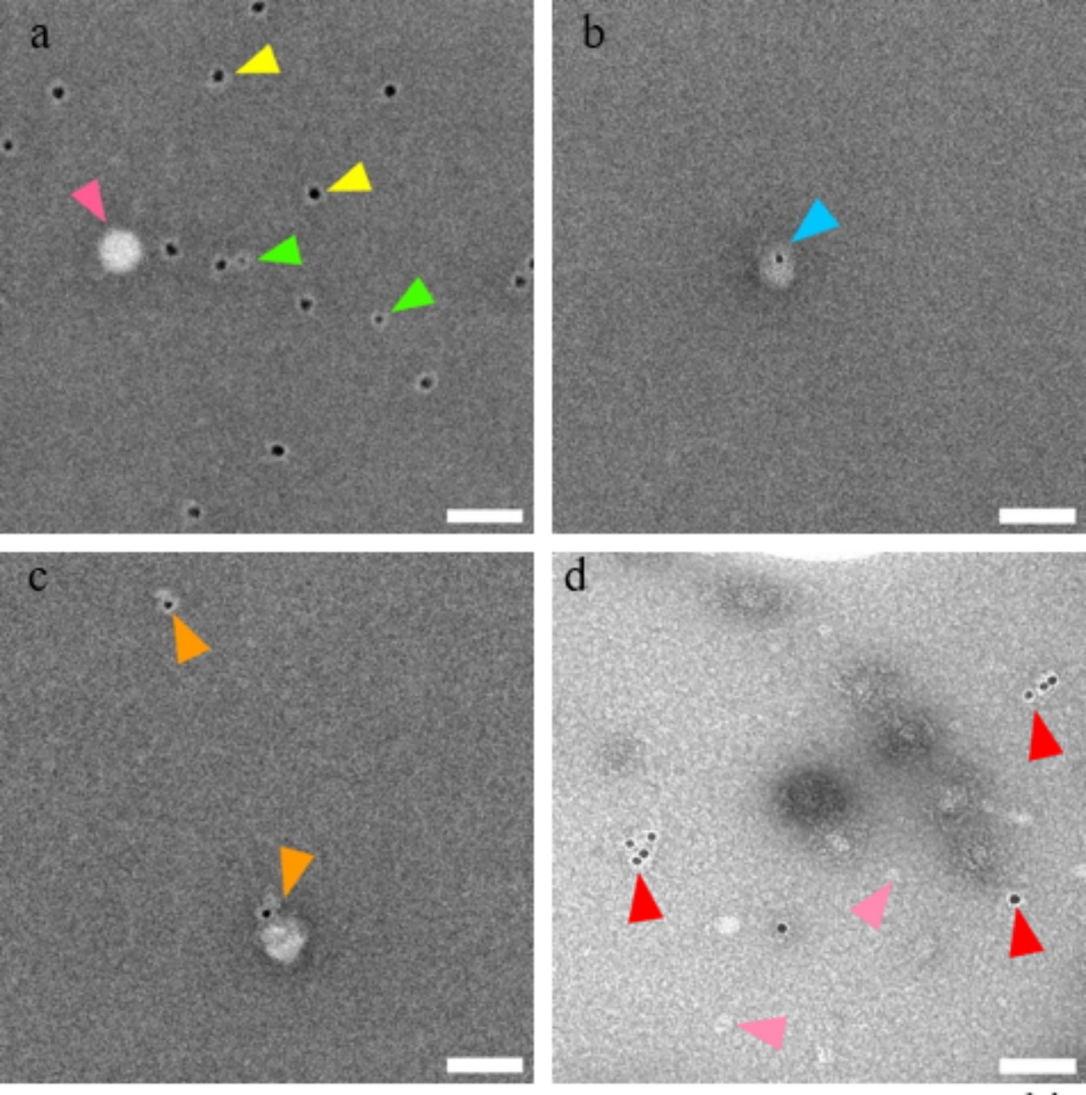
TEM analysis of extracellular vesicles in trapped CSF. This figure shows TEM images resulting from the immunogold labelling technique to visualize EVs isolated from CSF. (**a**) EVs have been immunolabeled with primary antibodies against CD9 and ATP1A3, followed by 15 nm and 10 gold-conjugated secondary antibodies, with yellow arrows highlighting CD9 positive, green ATP1A3 positive stained EVs. The pink arrow indicates non-labelled EVs. The scale bar represents 100 nm. (**b**) EVs have been immunolabeled with primary antibodies against CD81 followed by 10 nm gold-conjugated secondary antibodies, with a blue arrow highlighting CD81 positive. The scale bar represents 100 nm. (**c**) EVs have been immunolabeled with primary antibodies against CD63 followed by 10 nm gold-conjugated secondary antibodies, with orange arrows highlighting CD63 positive. The scale bar represents 100 nm. (**d**) EVs have been immunolabeled with primary antibodies against GLT-1 followed by 10 nm gold-conjugated secondary antibodies, with red arrows highlighting CD81 positive. The pink arrow indicates non-labeled EVs. The scale bar represents 100 nm. Corresponding negative control images with secondary antibodies revealed no specific binding, data is not presented.

To assess the presence and spatial distribution of EVs from CSF samples isolated by our trapping method, TEM was employed alongside immunogold labeling (Fig. 5). Targeting the EV-specific marker CD9 and brain-derived marker ATP1A3, we utilized primary antibodies against CD9^34^ and ATP1A3^54^, which were then visualized using secondary antibodies conjugated with gold particles measuring 15 and 10 nm, respectively (Fig. 5a). This allowed for specific visualization of the EVs. The analysis included samples from the trapped CSF specimens, which underwent this comprehensive visualization protocol. Furthermore, **Supplementary Fig. 2**, demonstrates the lack of unspecific bindings.CD81 positive EVs are depicted in (Fig. 5b**)**, using secondary antibodies conjugated with gold particles measuring 15 nanometers. CD63 positive EVs are depicted in (Fig. 5c**)**, using secondary antibodies conjugated with gold particles measuring 15 nanometers. An astrocyte-specific EVs maker GLT-1 positive EVs are depicted in (Fig. 5d**)**, using secondary antibodies conjugated with gold particles measuring 15 nanometers^55^.

### Extracellular vesicle lysis methods

**Fig. 6:**
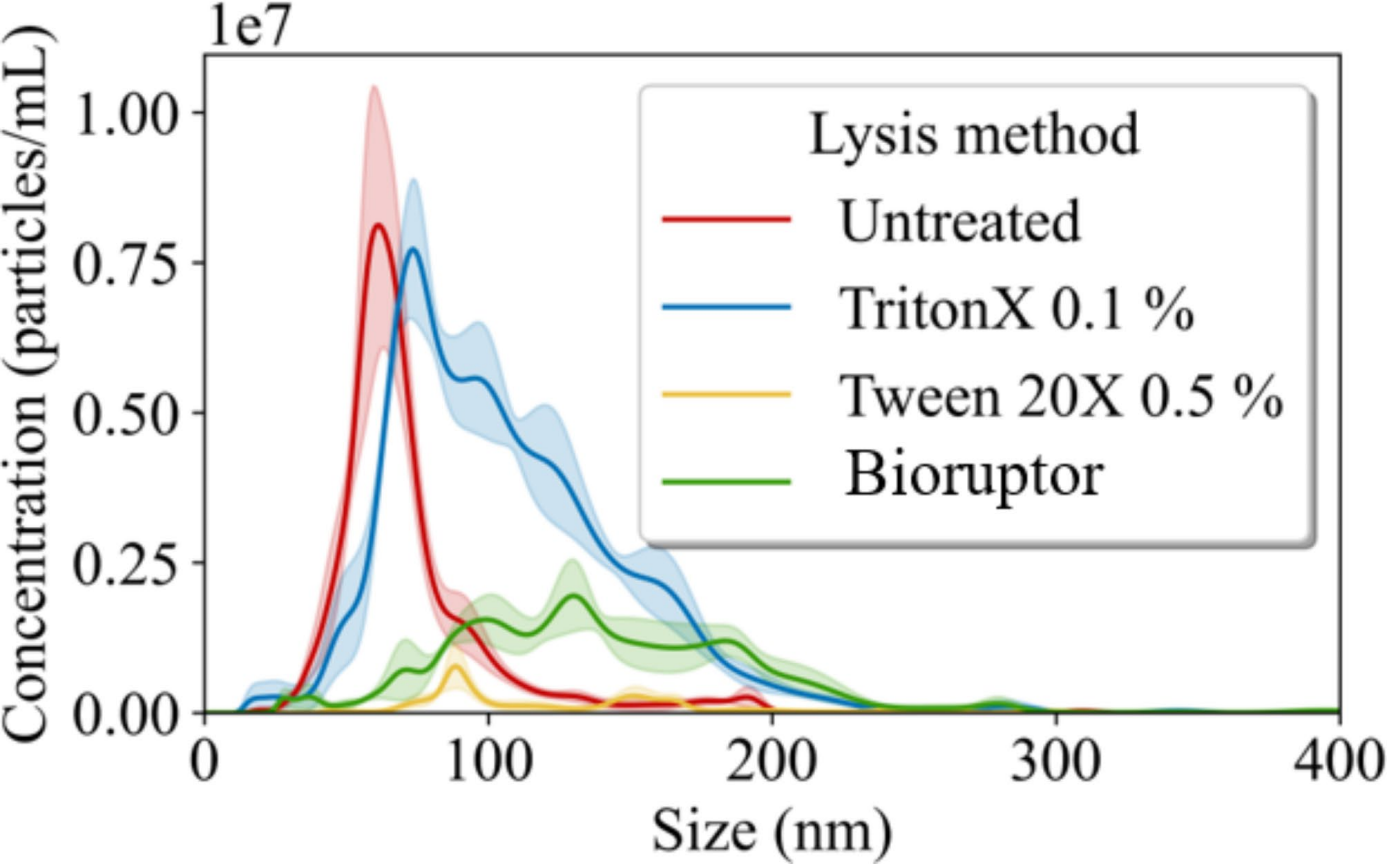
Evaluation of EVs Lysis Methods from Trapped CSF. This analysis was conducted on EVs isolated from the CSF of a healthy control subject, following washing at a flow rate of 200 µl/min. Illustrates particle distribution profiles derived from NTA after applying various EV lysis techniques. The methodologies tested included Triton X at 1% 25 min incubation (Blue), Tween 20X at 0.5% (Yellow), and a mechanical lysis method, Bioruptor, performed for 20 cycles (alternating 30 s off and on) (Green). Each lysis method was assessed for its impact on particle distribution, with results indicating distinctive effects from each treatment compared to the control sample (Red).

To prepare for the assay we wanted to make sure that trapped EVs release enough cargo for MSD to be able to measure the encapsulated tau. We have compared multiple lysis methods, Bioruptor Plus (Diagenode), Triton-X, and Tween. The lysis efficacy of each method was validated using NTA (Fig. 6) to measure the particle size distribution following treatment compared to an untreated reference, which represents the intact EV distribution. Tween-20X treatment at 0.5%, demonstrated a significantly higher reduction in EV concentration after only 5 min and was superior to Triton-X 0.1% (even after 25 min) and the Bioruptor (20 min at 4 °C). Tween is a gentle, non-ionic detergent which doesn’t have issues with denaturing proteins like Triton-X. Therefore, we used the Tween-20X treatment at 0.5% going forward.

### Aspiration volume

**Fig. 7:**
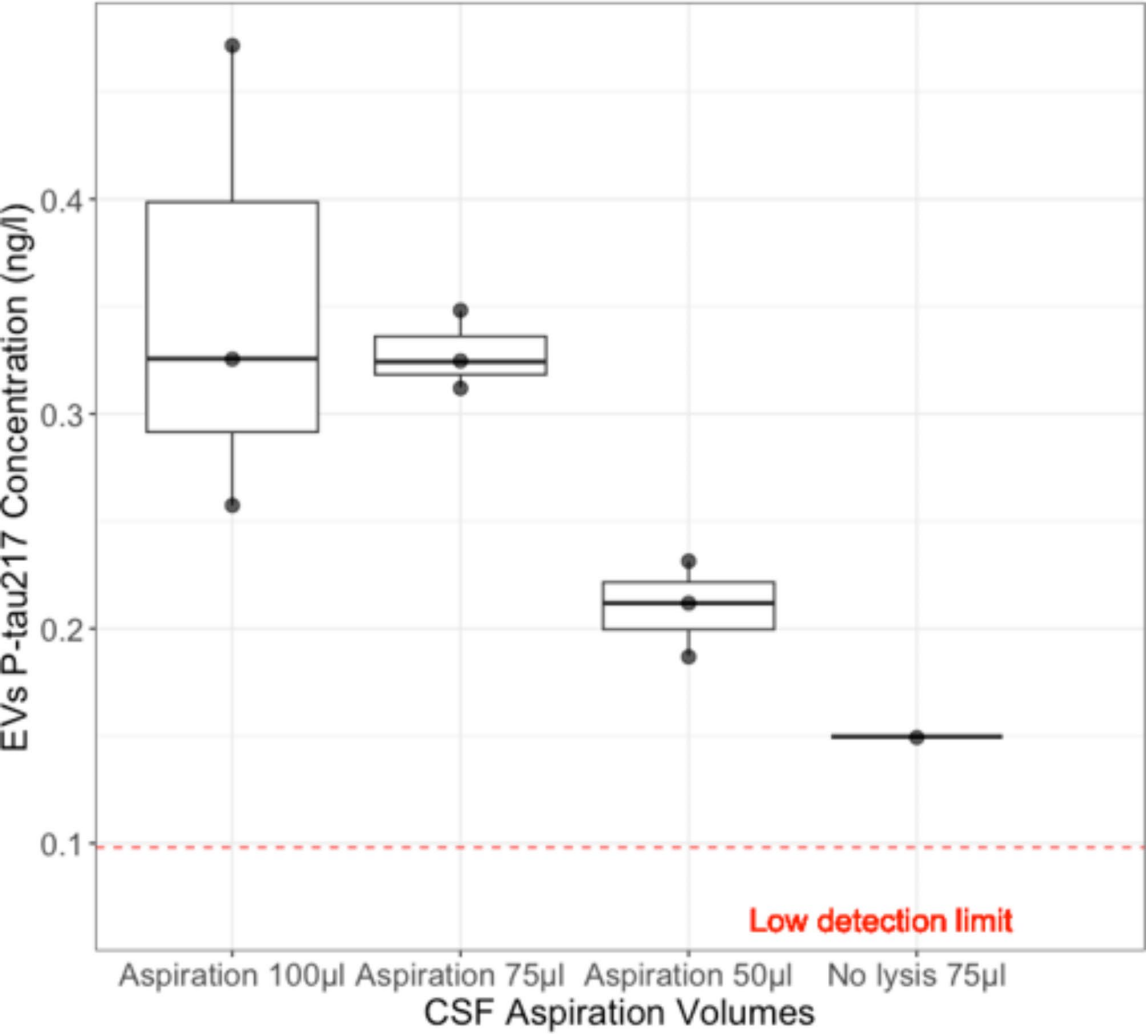
Assessment of P-tau217 levels from lysed EVs from trapped CSF samples using meso scale discovery (MSD). This assay quantifies P-tau217 in EVs isolated from the CSF of an AD patient (*N* = 1) from the preliminary group, from three different MSD plates. Each sample underwent two trapping cycles at specified aspiration volumes to ensure consistency. CSF samples were aspirated at volumes set at 100 µl, 75 µl, and 50 µl for acoustic trapping and then EVs were lysed using 0.5% Tween. In the final column is the signal from a sample trapped from 75 µl but without lysis. The assay’s sensitivity was calibrated to detect P-tau217 at a minimum of twice the concentration of the lowest standard, ensuring reliable low-level detection. The low-level detection limit is average *N* = 3.

We performed preliminary experiments to optimize the CSF aspiration volume for the EV P-tau217 assay, by lysis EVs and using P-tau217 concentration as the outcome measure (Fig. 7). The highest P-tau217 concentration was observed at 100 µl, as no significant increase in P-tau217 concentration was noted at 200 µl (data not shown). This indicates that 75 µl is the optimal volume of CSF to aspirate for effective EV trapping, in terms of P-tau217 levels inside EVs. Therefore, for the rest of the experiments, we used a 75 µl aspiration volume. Although we did not quantify the EVs in this experiment, these findings suggest this volume could be close to the maximum capacity for EV trapping. Additionally, the increase in P-tau217 following lysis supports the notion that P-tau217 is transported within EVs. The non-lysed EVs from the 75 µl trapped sample had only 0.15 ng/l of P-tau217, whereas lysis increased the P-Tau217 concentration to 0.33 ng/l.

### Content of EVs: P-tau217

**Fig. 8:**
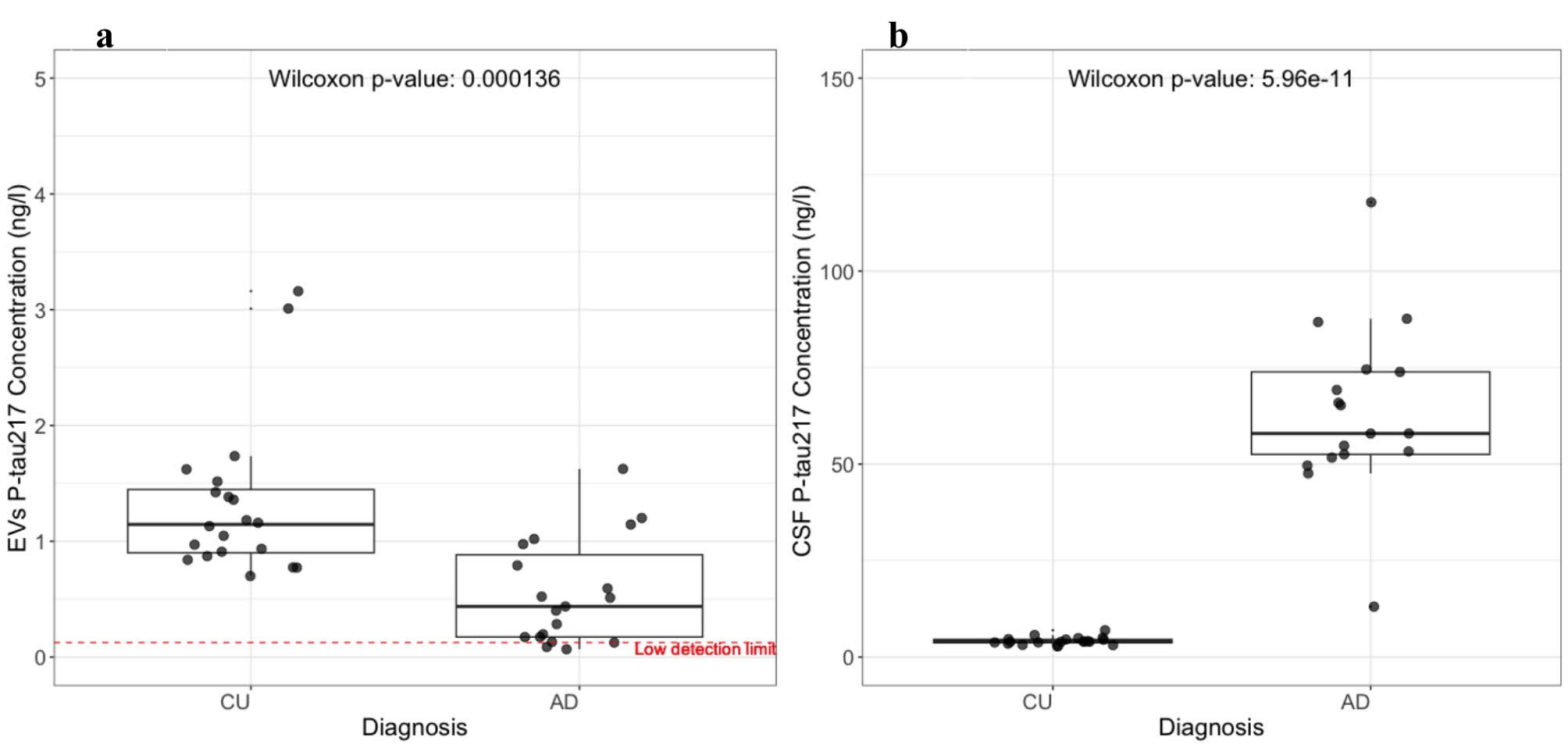
P-tau217 in CSF and EVs. (**a**) Meso Scale Discovery assay of P-tau217 from trapped EVs from the same CSF samples. Aspiration of 75 µl at 200 µl 1/m. EVs were lysed with tween 0.5%. The red dashed line is a low detection limit (twice the lowest value on the calibration standard). A Wilcoxon rank-sum test indicates a statistically significant difference between the groups for both CSF and EVs but with opposite directions. (**b**) Meso Scale Discovery assay of P-tau217 from CSF of AD (*N* = 20) and CU (*N* = 20) samples.

We next analyzed P-tau217 (Fig. 8) in EVs and CSF. P-tau217 concentrations in lysed EVs were lower in EVs trapped from patients with AD compared to CU individuals (Fig. 8a). with a p-value of 0.0001. The Spearman test has shown no correlation for either sex or age for both trapped and CSF samples, for P-tau217 levels. When comparing P-tau217 distributions in CSF to those in lysed EVs, we see an opposing trend, with higher P-tau217 levels in AD compared to CU (Fig. 8b). We observed that 15% of the AD group had values for P-tau217 in EVs that were below the low detection limit, compared to 0% in the CU group. Additionally, **Supplementary Fig. 3**, shows that CSF Aβ42/Aβ40 ratio was not associated with trapped P-tau217 levels, within diagnostic groups.

### Content of EVs: P-tau181

**Fig. 9:**
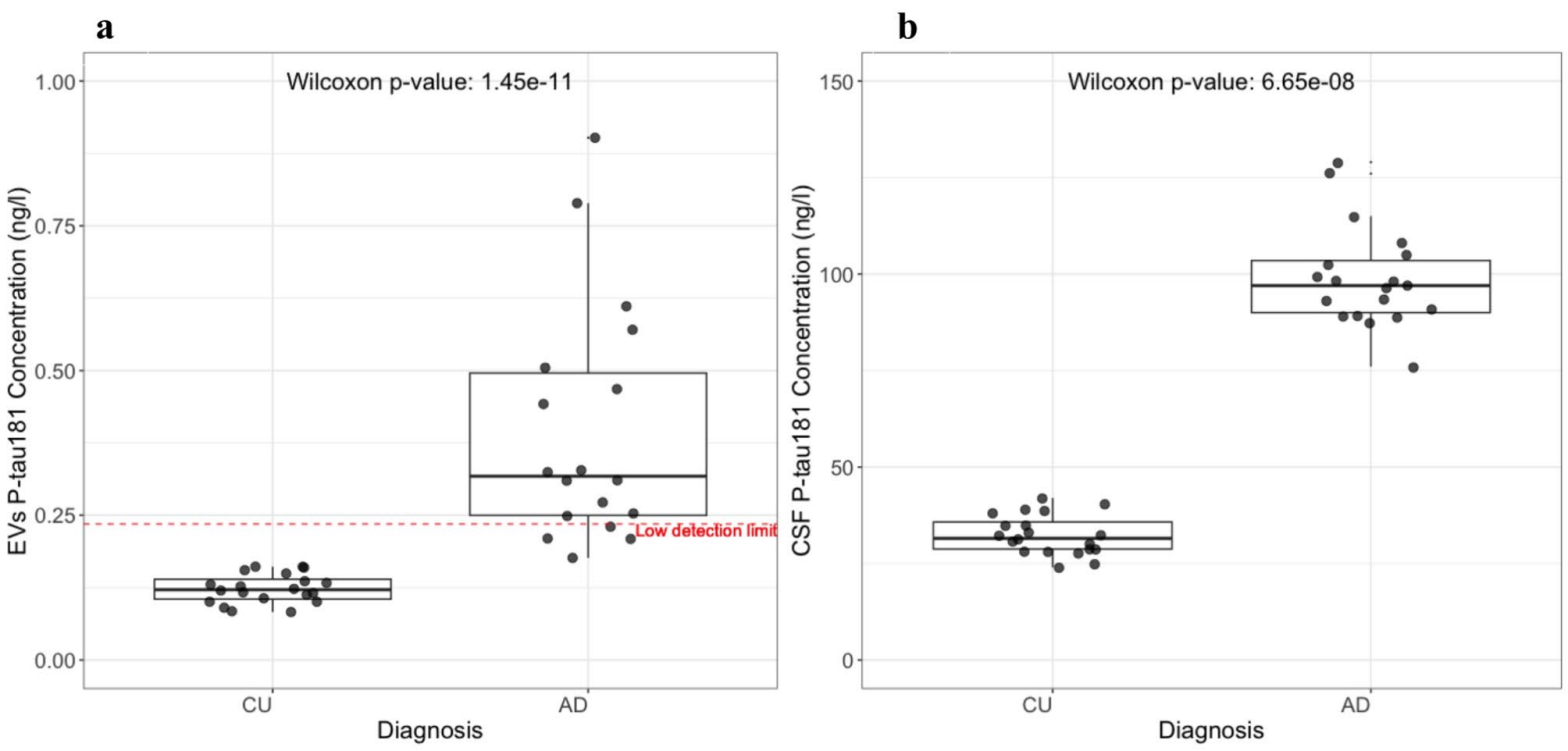
P-tau181 in CSF and EVs. Meso Scale Discovery assays of P-tau181 from CSF of AD (*N* = 20) and CU (*N* = 20) samples. (**a**) Meso Scale Discovery assay of P-tau181 from trapped EVs isolated from the 40 patient CSF samples. Aspiration of 75 µl at 200 µl l/m. EVs were lysed with tween 0.5%. The red dash line is a low detection limit (twice the lowest value of a standard). The Wilcoxon rank-sum test indicates a statistically significant difference between the groups for both CSF and EVs, with a similar correlation trend (**b**) Meso Scale Discovery assay of P-tau181 from unprocessed CSF of the same participants.

When quantifying P-tau181 levels within lysed EVs, we observed a statistically significant higher level in AD compared to CU (Fig. 9a), similar to what was seen in unprocessed CSF (Fig. 9b). There was no association between P-tau181 levels and either sex or age, for P-tau181 in either EV or CSF. We observed that 20% of the AD group had values for P-tau181 in EVs that were below the low detection limit, compared to 100% in the CU group Additionally, **Supplementary Fig. 3**, shows that the CSF Aβ42/Aβ40 ratio was not associated with trapped P-tau181 levels, within diagnostic groups.

### Content of EVs P-tau181/P-tau217 ratio

**Fig. 10:**
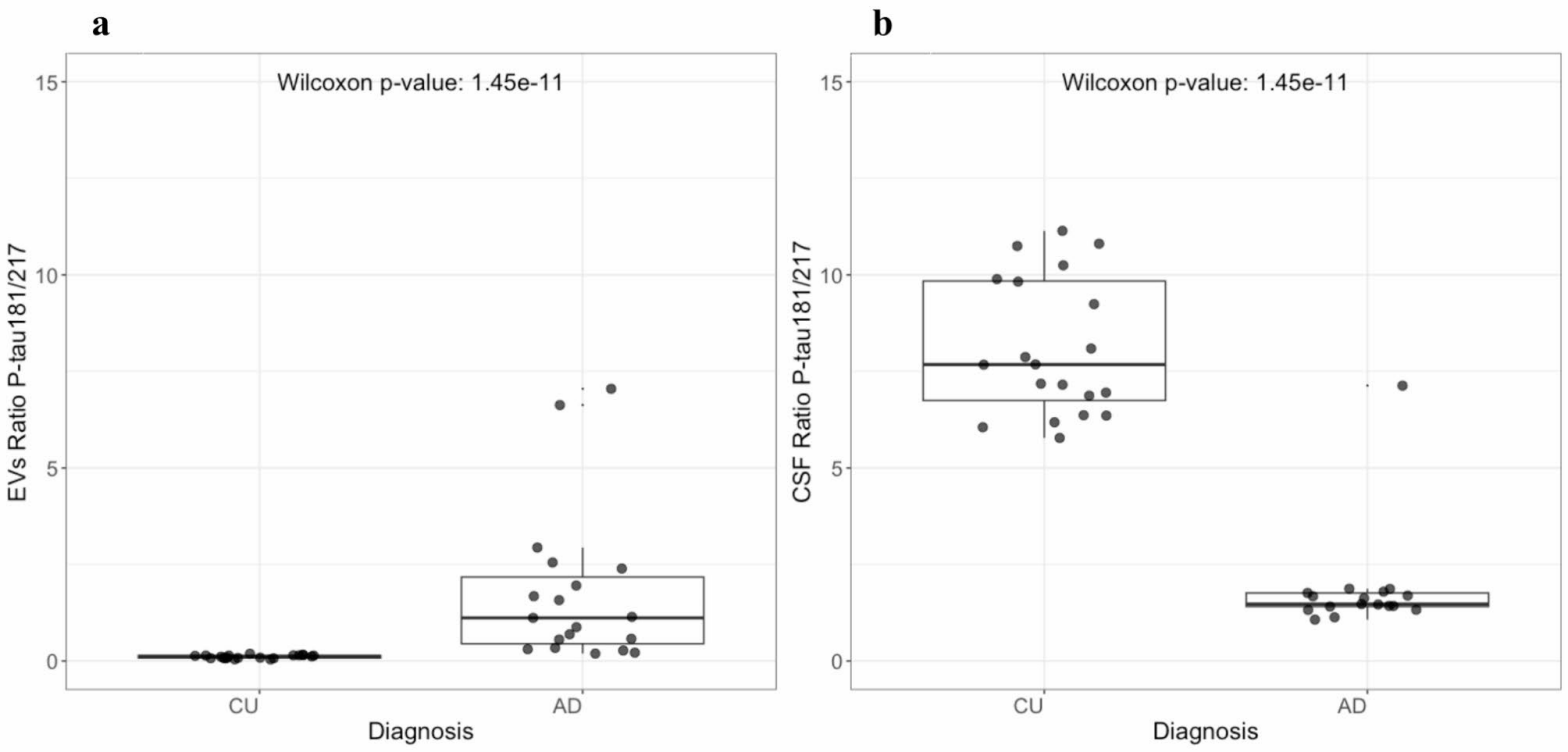
Biomarker ratios. We calculated ratios of P-tau181 and P-tau217 levels in EVs and CSF of AD (*N* = 20) and CU (*N* = 20) samples. (**a**) Ratio of EVs P-tau181/217. A Wilcoxon rank-sum test indicates a statistically significant difference between the two patient groups in both CSF and EVs, with an opposite correlation. (**b**) Ratio of CSF P-tau 181/217.

In sum, we found differing trends for P-tau cargo of EVs from AD compared to CU patients in the cohort, for P-tau181 (increased) and P-tau217 (reduced). To investigate the extent of this correlation in AD versus CU, we expressed the ratio of P-tau181 to P-tau217 abundance, which was markedly increased in trapped EVs in AD compared to CU individuals, (Fig. 10a). By contrast, the CSF P-tau181/217 ratio was significantly decreased, (Fig. 10b).

### Contents of EVs in relation to other characteristics of EVs

While differences in the ratio of P-tau181/217 between AD and CU groups were noted, the isolated EV numbers and EV mean size were not found to significantly differ between these groups, (**Supplementary Fig. 1**). Furthermore, there was no significant correlation between the number of EVs and the concentrations of P-tau217 and P-tau181, (**Supplementary Fig. 4**), or number of EVs and participants age, (**Supplementary Fig. 6**). Additionally, we explored the potential influences of demographic factors on these biomarker levels and found no significant variances attributable to sex or age within our study population on trapped EVs and CSF P-tau181 and P-tau217 levels, (**Supplementary Figs. 5 and 7**).

### Reproducibility of EV trapping

**Fig. 11:**
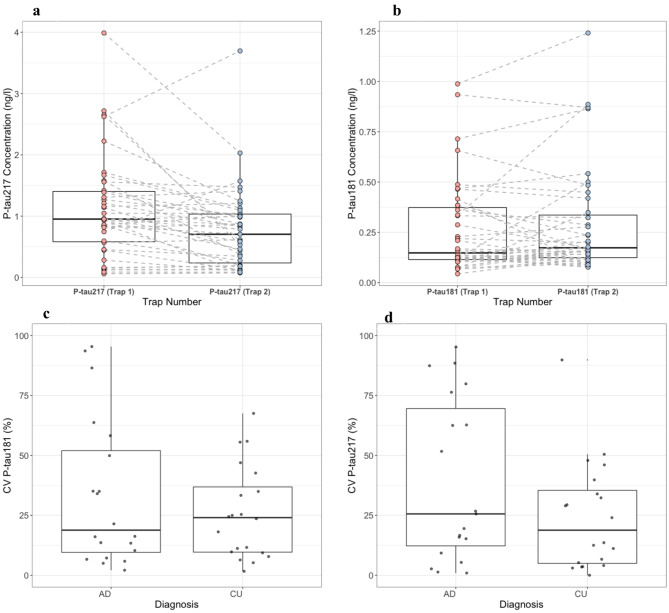
Reproducibility tests of EV contents. Box plots (**a**) and (**b**) comparison of trap 1 and trap 2 for P-tau181 and P-tau217 measurements in EVs. The dotted line connects the same individuals’ samples in two traps. (**c**) and (**d**) cumulative variance (CV) % for trap 1 and trap 2 of P-tau181 and P-tau217 concentration measurements.

To validate the reproducibility of our method of isolation of EVs, we processed samples in duplicates (two separate isolation of EVs performed one after another) within the same plate readout runs to ensure the reliability of our MSD assay. Figure 11a and b illustrate a comparative analysis between the first and second trapping sessions, highlighting the consistency in a majority of biomarker capture from identical patient samples. Additionally, Fig. 11c and d present the mean coefficient of variation for both P-tau181 and P-tau217 across the two trapping runs. This dual-level repetition—both within and between assay runs—demonstrates a reasonable level of stability and reproducibility in our EVs isolation and analysis techniques, although the relatively high CV values suggest some variability that warrants consideration.

## Discussion

This is the first demonstration of acoustically trapping of EVs from CSF to our knowledge, and subsequent quantification of EV encapsulated P-tau proteins from patient samples. By appropriate detergent lysis, we were able to quantify P-tau181 and P-tau217 levels from within the CSF-derived EVs. Furthermore, we identified cohort-level differences in the EV cargo levels of P-tau181 and P-tau217 between patients with AD and those who were CU. This supports the hypothesis that EVs may play a role in AD and that this could be observed in the transport of specific variants of P-tau. Levels of P-tau181 and P-tau217 in AD patients’ plasma and CSF start to increase during the pre-symptomatic phase when cortical Aβ fibrils emerge and before insoluble tau tangles can be detected with tau-positron emission tomography^48^. Notably, P-tau217 is more strongly correlated with brain Aβ than with tau aggregation^56–58^. Tau proteins, identified in interstitial fluids and CSF preceding neurodegeneration, may be released via an active secretory process^59^. While the majority of tau is secreted in a free form, a significant portion has been attributed to EVs, including exosomes^60,61^. Understanding the mechanisms of tau propagation between cells and its role under physiological conditions is critical for improving disease monitoring and developing targeted therapies. Pathological tau seeds can transfer between co-cultured cells, leading to the templated recruitment of native tau proteins to form new pathogenic seeds – a phenomenon observed in various in vitro and in vivo models^62–64^. EVs may serve as vehicles for this transfer, being secreted by parental cells and internalized by recipient cells, facilitated by specific markers that promote uptake^65^. The fusion of EVs with cell membranes may occur through endocytosis^66,67^, with smaller EVs being more readily internalized than their larger counterparts^68^.

The analysis of lysed EVs (an average of two technical replicates) revealed higher levels of P-tau181 in the AD group relative to the CU group, mirroring the trend observed in CSF P-tau levels (Fig. 8**)**. Remarkably P-tau217, presented higher levels in trapped EVs from CU, whilst it was elevated in AD CSF of the same cohort (Fig. 9**)**. The trapped EVs ratio of P-tau 181/217 was notably higher in the AD group than in the CU group. Whereas conversely, the CSF ratio of P-tau181/P-tau217 was higher in the CU group (Fig. 10**)**. This could indicate a difference in the expression or clearance rates of specific variants of P-tau. Another study suggests that EVs-mediated secretion of P-tau may play a significant role in the abnormal processing of tau and in the origin of elevated CSF tau in early AD^17^. Our study also explored the potential influences of demographic factors on these biomarker levels and found no significant variances attributable to sex or age within our study population on trapped EVs and CSF P-tau181 and P-tau217 levels (**Supplementary Figs. 5 and 7**). This absence of demographic effects supports the hypothesis that the observed biochemical differences are more directly related to disease processes rather than age or sex-based physiological variations. Furthermore, Spearmen has shown that the CSF Aβ42/Aβ40 ratio does not affect trapped P-tau levels (**Supplementary Fig. 3**). There was also no significant correlation between the number of EVs and the concentrations of P-tau217 and P-tau181 (**Supplementary Fig. 4**). Additionally, although some differences in the P-tau181/217 ratio between the AD and CU groups were observed, the relationship influenced by the ratio of isolated EV number and EV mean size did not show a significant variation between the groups. This finding suggests that while EVs carry disease-relevant biomarkers, their absolute counts or size distributions do not necessarily reflect the pathological state, reinforcing the importance of focusing on biomarker content rather than exclusively using EV quantity or size for disease characterization. Despite intriguing findings related to EVs, CSF P-tau levels still provide a more distinct differentiation between CU and AD groups, making them a better indicator for monitoring AD pathology. Furthermore, recent developments in the use of high-performance blood tests in clinical practice for accurate AD diagnosis and AD-specific treatments^12^ make EV P-tau levels less interesting as a diagnostics tool, as using CSF is more invasive than plasma. However, EV findings may reveal mechanistic information on the pathology and spread of AD, which is needed for targeted therapies in the future.

NTA analysis of isolated EVs showed variability in EV concentrations, whilst the mean number of particles isolated was around 5 × 10^8^ and the mean EV size was 144 nm (Table 1; Fig. 4). There were no significant differences in the quantity or mean size of isolated EVs between AD and CU, contrary to other research done on EV cargo that found higher EV concentrations and smaller EV sizes in AD CSF^69^. The size and concentration of EVs can differ hugely between individuals, but the quantification method can also affect the measurements, which could play a role in this discrepancy. In our study, we relied on the continuous flow NTA where EVs flow through a microfluidic chip during measurement, whereas a static measurement was performed in the other study^69^ - where samples remain still in a chamber with no flow. Continuous flow measurements have been found to out-perform static measurements of this kind, showing lower variance in size and concentration distribution^70^. In our study, NTA has shown that the mean size of EVs is 144 nm in diameter, with no significant difference between AD and CU groups and more variation on an individual basis. Some of this variation could be due to the measurement limitations, but with current equipment EV size and concentration doesn’t appear to be a good diagnostic of AD, perhaps due to too many confounding factors or a limited cohort size. Meanwhile, TEM revealed that we have predominately 10–30 nm EVs, (Fig. 5 and **Supplementary Fig. 2**). TEM has been instrumental in analyzing the role of diminutive EV subsets like exomeres, whose biological role remains unknown but have been observed below 50 nm and carrying proteins involving metabolism^71^. Measurements of EV size distributions will be limited by the specifications of each tool. In the case of TEM, sometimes dehydration and shrinkage of EVs can occur in the sample preparation process^72^. NTA calculates hydrodynamic size distributions of particles in suspension. On the other hand, NTA is biased towards larger EV sizes since the detection relies on the refractive index and particle size being sufficient to scatter enough light to be detectable (limited to above ~ 50 nm for EVs). Larger vesicles may scatter light and hide smaller-sized EVs which makes the size distribution unreliable at high concentrations. To mitigate this, we diluted the samples to around 10^7^-10^9^ particles/ml.

Furthermore, TEM immunophenotyping of EVs was confirmed with a positive labeling of common tetraspanin markers, CD9, CD63, and CD81 which are generally considered classical EV markers using TEM (Fig. 5)^50^. Studies indicate that these markers are not equally expressed in all EVs but rather show heterogeneity that reflects the expression levels of their cell of origin, such as neuronal or glial cells^73^. Generally, in CSF, the CD81 positive EV levels are higher than CD9 positive EV levels, which are significantly higher than the CD63 positive EV levels^74^. However, in our study (Fig. 5) we qualitatively observed more small-size CD9 positive EVs, with slightly fewer CD63, which were larger in general. Meanwhile, we did not observe a significant number of CD81-positive EVs. Furthermore, we were able to confirm the cerebral origin of many EVs via ATP1A3 – a marker predominantly associated with brain-derived EVs that also expressed across various neuronal types and astrocytic glutamate transporter 1 (GLT-1)^54,75^. The pivotal role of EVs in glial-to-neuronal communication has gained recognition^76^. Microglial transcripts are associated with tissue Aβ and tau density^77^. EVs secreted by astrocytes are key factors in supporting neuronal functions^78^. Both microglia depletion and inhibition of EV production decreased tau propagation in experimental models^79,80^. Investigating if CSF EVs are released by activated microglia and astrocytes in response to AD pathology is crucial, as these EVs carry pro-inflammatory molecules, such as cytokines and chemokines, which can induce inflammatory responses in recipient cells, leading to neuronal damage and exacerbating AD pathology^81,82^.

During optimization, we focused on the P-tau levels. We demonstrated the presence of P-tau as cargo of EVs. We then explored the optimal lysis method, as detergents and their concentrations affect different EV subpopulations^83^. Tween 20 at a 0.5% concentration was effective for lysing EVs while maintaining the integrity of immunoassays targeting P-tau (Fig. 6). Additionally, optimizing aspiration volume for CSF preservation increased P-tau217 with tested aspiration volume up 200 µl. We saw no benefits above 75 µl aspiration volume (Fig. 7). While we cannot definitively claim it has reached its maximum capacity, the results provide strong evidence that it is likely close to it, as P-tau levels realized from EVs do not increase. Furthermore, the increase in P-tau following lysis indicates that P-tau is transported within the EVs. Overall, this microfluidics-based automated methodology exhibits efficient and reproducible handling of very small volumes (as low as 25 µl) Being able to recover EVs from such small volumes allowed us to perform multiple analyses (trapping up to 6 technical replicates) on the same aliquot. To recover EVs from CSF via ultracentrifugation or even size exclusion chromatography would require substantially larger volumes. Getting access to CSF is invasive and hard to come by, hence these experiments are rarely done. However, acoustic trapping makes it possible to access EV content from CSF in a more efficient way. The rapid nature of this purification technique also makes scaling up to handle more samples possible, as we have demonstrated by acoustically trapping EVs from aliquots of CSF of 40 patients with 5–6 repeats. Ultimately, acoustic trapping is a technique designed for low-volume purification– the single node trapping capillary used here has a total volume of around 16 µL and our protocol is run with washing buffer such that there is no dead volume in the surrounding tubing and all of the samples gets exposed to the acoustic field. The more commonplace methodologies, ultracentrifugation, and size-exclusion chromatography would fail to process 6 technical replicates from the patient sample volumes available.

It is also important to address the limitations of the study. First, the concentration of EVs measured by NTA in our trapped samples appears to not paint the full picture since TEM revealed many EVs smaller than 50 nm, below the detection limit of NTA. The heterogeneous nature of the EVs we isolate by acoustic trapping can be seen as both an advantage and a disadvantage. It limits our knowledge of which subpopulation of EVs which carry the P-tau cargo that we identified. However, it also allows a fuller picture of the EVs present in native CSF than an immunoaffinity isolation technique would allow. Another limitation of this work is that it relies on the MSD detection sensitivity, for example, the healthy controls were below the detection limit, so it is not clear whether P-tau181 is carried in EVs at low concentration in the CU group or if it is only free-floating. We analyzed a sample size of 20 AD patients and 20 CU individuals, which, although sufficient to detect significant differences between the groups in this study, might limit the power to generalize the findings broadly. One notable challenge in this work is the potential variability for the EV isolation and characterization methods. The sensitivity limitations of detecting P-tau within EVs complicate the accurate quantification of low concentrations in some study participants. Furthermore, multiple biological factors can hypothetically affect EVs. We found no statistical correlation (within diagnostic groups) between EV content and age, sex, or MMSE scores, but we cannot exclude that there may be other confounding factors that could hypothetically affect the levels of P-tau in EVs. In summary, our findings suggest that small EVs with confirmed cerebral origin via ATP1A3 and GLT-1 expression can be isolated using acoustic trapping from the CSF of AD and CU individuals. We found intriguing differences in levels of different P-tau variants in EVs, providing opportunities for new biomarker-related discoveries. Further characterization, e.g., for microglia-derived EVs implicated in tau spread in AD, is needed. Future studies will investigate P-tau levels in specific EV subpopulations to enhance our understanding of tau propagation in the brain in AD. We will also expand sample sizes to improve the generalizability and reliability of P-tau detection in EVs.

## Electronic supplementary material

Below is the link to the electronic supplementary material.

Supplementary Material 1

Supplementary Material 2

## Data Availability

The data supporting the findings of this study are provided within the manuscript and its supplementary information files. Additional datasets generated and/or analyzed during the current study are available from the corresponding author upon reasonable request.
