# Supplementary material for: Phosphorylated tau in cerebrospinal fluid-derived extracellular vesicles in Alzheimer’s disease: a pilot study
Source: Sci Rep. 2024 Oct 25;14:25419. doi: 10.1038/s41598-024-75406-0 (PMC11511998; doi:10.1038/s41598-024-75406-0)
Supplement: Supplementary file 1 — Supplementary Material 1 [file 41598_2024_75406_MOESM1_ESM.docx]

**Phosphorylated Tau In Cerebrospinal Fluid-Derived Extracellular Vesicles In Alzheimer’s Disease: A Pilot Study**

*Roman* ***Sattarov****^1^****^*^****, Megan* ***Havers****^2*^, Camilla* ***Orbjörn****^1^, Erik* ***Stomrud*** *^1,3,4^, Shorena* ***Janelidze****^1,4^, Thomas* ***Laurell****^2^, Niklas* ***Mattsson-Carlgren****^1,4,5*^*

*Affiliations:*

*1 Clinical Memory Research Unit, Department of Clinical Sciences Malmö, Lund University, Lund, Sweden.*

*2 Department of Biomedical Engineering, Lund University, Lund, Sweden*

*3 Memory Clinic, Skåne University Hospital, Malmö, Sweden*

*4 Wallenberg Center for Molecular Medicine, Lund University, Lund, Sweden*

*5 Department of Neurology, Skåne University Hospital, Lund, Sweden*

*Roman* ***Sattarov*** *(*[Roman.Sattarov@med.lu.se](mailto:Roman.Sattarov@med.lu.se)*)*

*Megan* ***Havers*** *(Megan.Havers@bme.lth.se)*

*Camilla* ***Orbjörn*** *(*[*Camilla.Orbjorn@med.lu.se*](mailto:Camilla.Orbjorn@med.lu.se)*)*

*Erik* ***Stomrud*** *(Erik.Stomrud@med.lu.se)*

*Thomas* ***Laurell*** *(*[Thomas.Laurell@bme.lth.se](mailto:Thomas.Laurell@bme.lth.se)*)*

*Shorena* ***Janelidze*** *(*[*Shorena.Janelidze@med.lu.se*](mailto:Shorena.Janelidze@med.lu.se)*)*

*Niklas* ***Mattsson-Carlgren*** *(*[Niklas.Mattsson-Carlgren@med.lu.se](mailto:Niklas.Mattsson-Carlgren@med.lu.se)*)*

*Correspondence to R.S. ([Roman.Sattarov@med.lu.se](mailto:Roman.Sattarov@med.lu.se)), M.H. ([Megan.Havers@bme.lth.se](mailto:Megan.Havers@bme.lth.se)) and N.M.C ([Niklas.Mattsson-Carlgren@med.lu.se](mailto:Niklas.Mattsson-Carlgren@med.lu.se))

**Supplementary Figures**


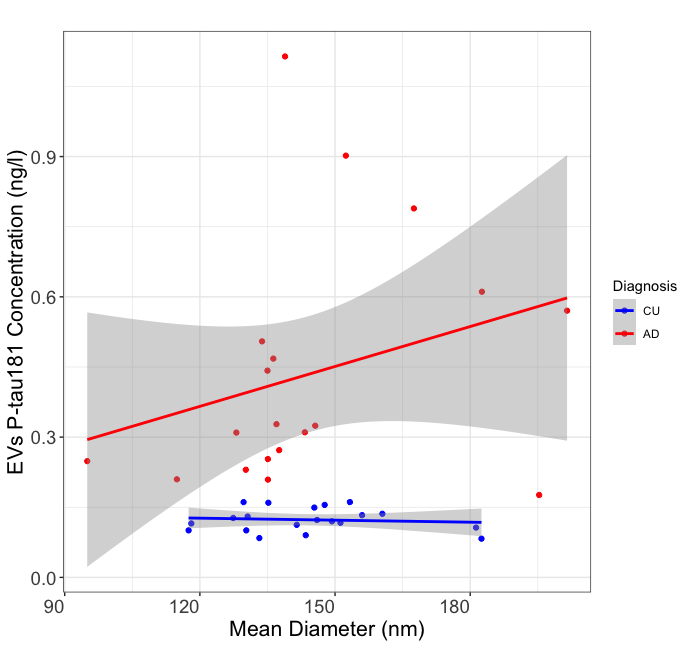

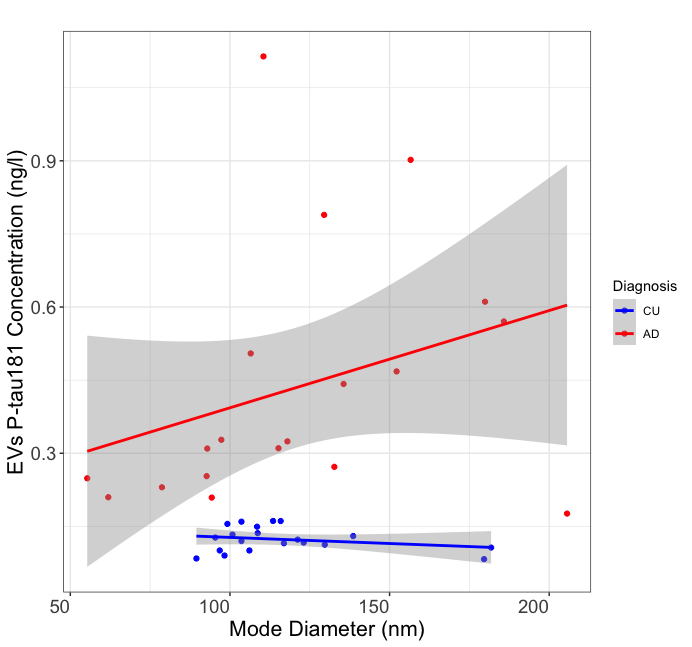

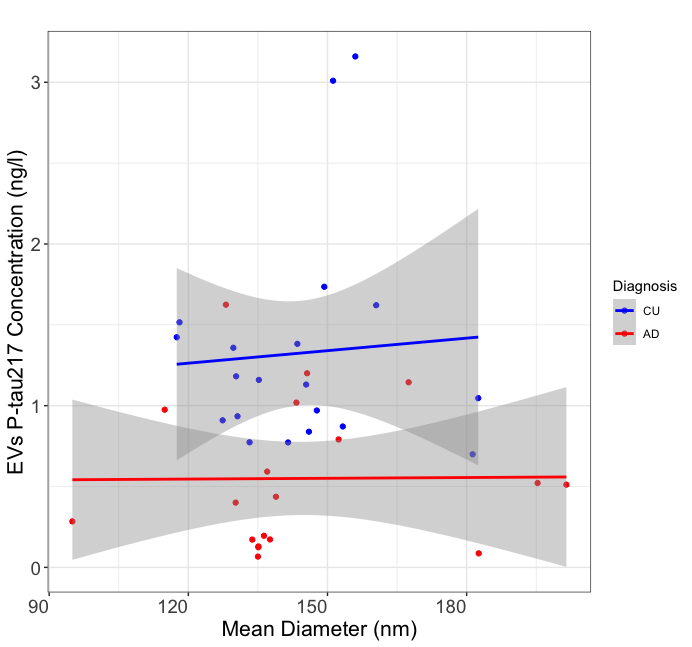

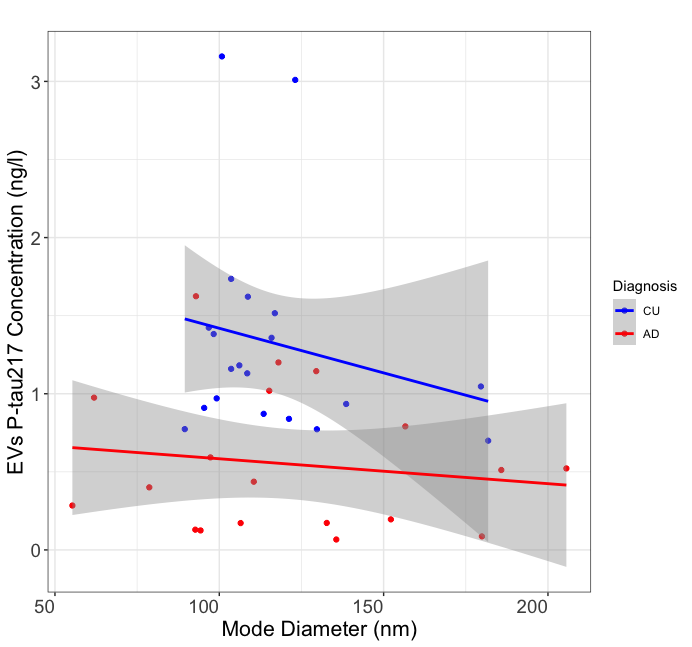


**a**

**b**

**c**

**d**

**Supplementary Figure 1**. **Correlation analysis between nanoparticle tracking analysis (NTA)** **-derived EV concentrations and P-tau217 and P-tau181 levels.** This scatter plot incorporates a linear model to illustrate the relationship between the mean diameter and mode (nm) of EVs, as measured by NTA, in trapped samples from Alzheimer’s disease (AD) patients (n=20) and cognitive unimpaired (CU) (n=20) (**a**) Mean size and P-tau181 concentration levels (ng/l) (**b**) Mean size and P-tau217 concentration levels (ng/l). **(c)** Mode of EVs size distribution P-tau181 concentration levels (ng/l) (**d**) Mode of EVs size distribution P-tau217 concentration levels (ng/l). Correlations were tested with Spearman’s rank correlation test, within the diagnostic groups. All correlations were non-significant (P>0.05).


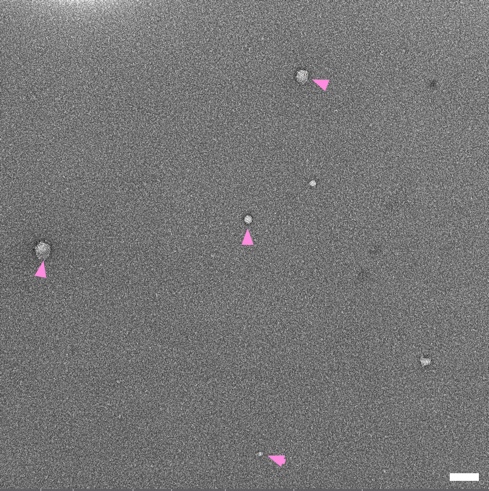


**Supplementary Figure 2. A negative staining from TEM analysis of extracellular vesicles in trapped CSF**. This figure shows TEM images with EVs incubated with only 15 and 10 nm gold-conjugated secondary antibodies (goat anti-mouse and goat anti-mouse, respectively) to check for unspecific binding in the absence of primary antibodies. The pink arrow indicates non-labeled EVs. The scale bar represents 200 nm.


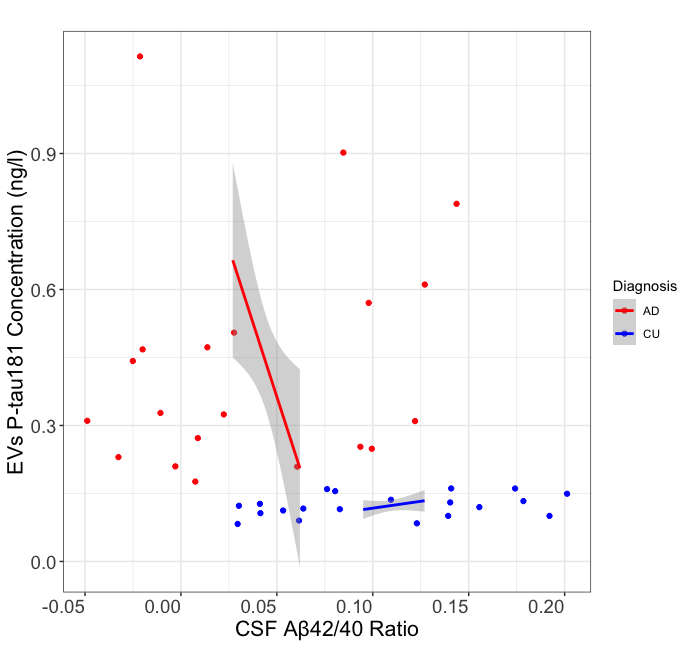

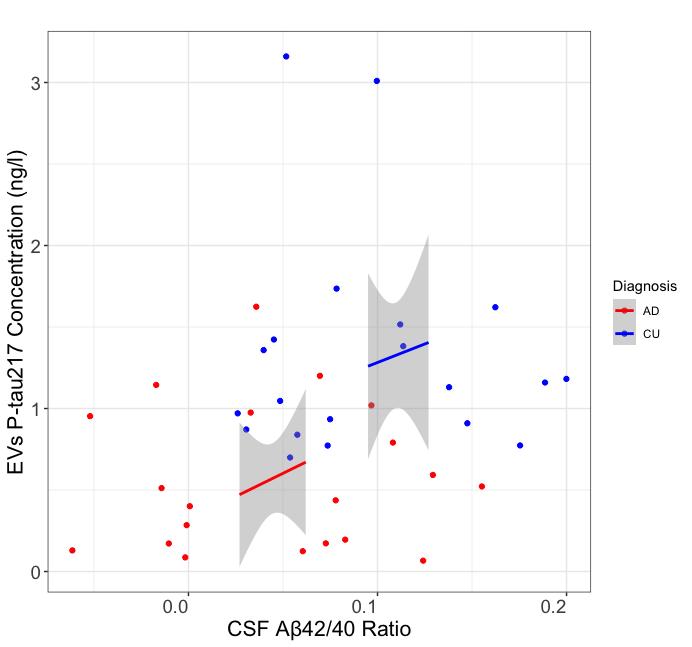


**a**

**b**

**Supplementary Figure 3. Scatter plot analysis of CSF Aβ42/Aβ40 ratio trapped EVs P-tau181 and P- tau 217 levels. (a)** Scatter plot of CSF Aβ 42/40 ratio against trapped P-tau181, with regression lines for each diagnosis group Alzheimer’s disease (AD) and cognitive unimpaired (CU). The model includes an interaction term between average trapped P-tau181 and Diagnosis. (**b**) Scatter plot of CSF Aβ42/40 ratio against average trapped P-tau217, with regression lines for each diagnosis group AD and CU. Samples from AD patients (n=20) and CU (n=20). Correlations were tested with Spearman’s rank correlation test, within the diagnostic groups. All correlations were non-significant (P>0.05).

**Supplementary Figure 4.** **Correlation analysis between nanoparticle tracking analysis (NTA) -derived EV concentrations and P-tau217 and P-tau181 levels**. This scatter plot incorporates a linear model to illustrate the relationship between the number of EVs, as measured by NTA, in trapped samples from Alzheimer’s disease (AD) patients (n=20) and cognitive unimpaired (CU) (n=20) and P-tau levels (**a**) EVs number and P-tau181 concentration levels (ng/l) (**b**) EVs number and P-tau217 concentration levels (ng/l). Correlations were tested with Spearman’s rank correlation test, within the diagnostic groups. All correlations were non-significant (P>0.05).


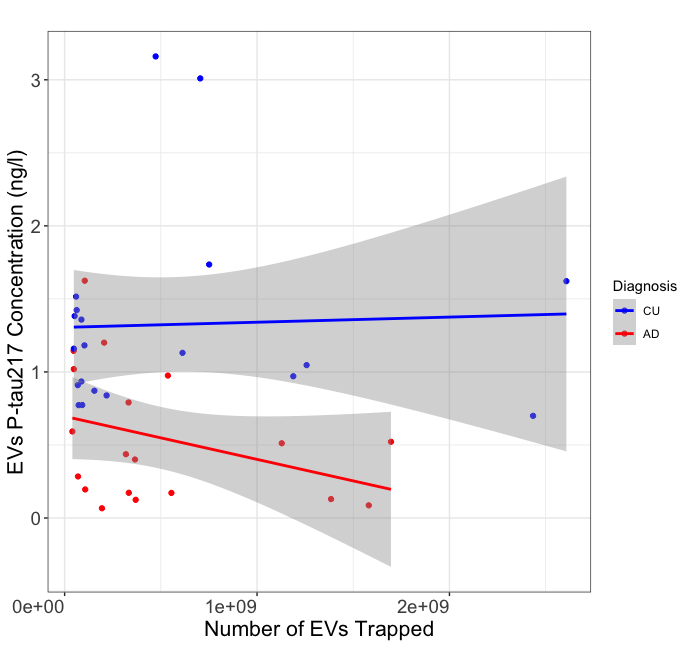

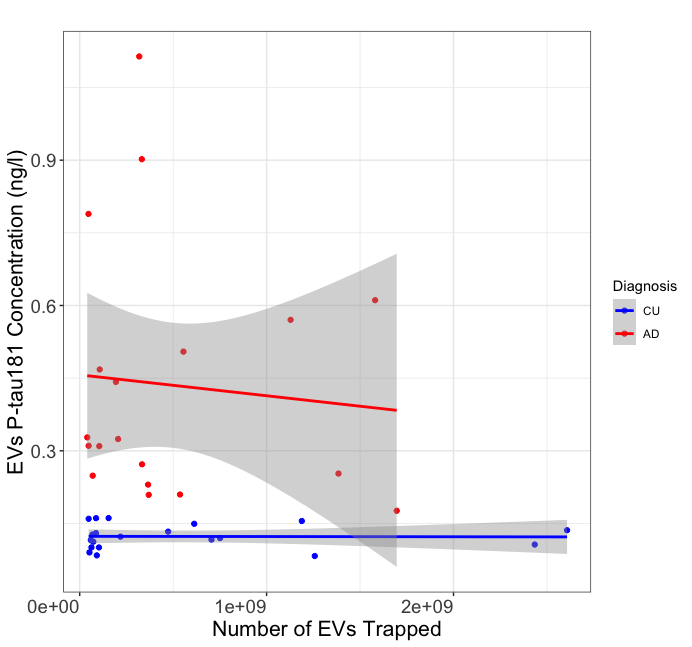


**a**

**b**

**Supplementary Figure 5.** **Correlation analysis between Age and EV concentrations of P-tau217 and P-tau181 levels**. This scatter plot incorporates a linear model to illustrate the relationship between Age and P-tau concentration levels in trapped samples and CSF from Alzheimer’s disease (AD) patients (n=20) and cognitive unimpaired (CU) (n=20) (**a**) Trapped EVs P-tau181 concentration levels (ng/l) (**b**) Trapped EVs P-tau217 concentration levels (ng/l) **(c)** CSF P-tau181 concentration levels (ng/l) **(d)** CSF P-tau217 concentration levels (ng/l). Correlations were tested with Spearman’s rank correlation test, within the diagnostic groups. All correlations were non-significant (P>0.05).


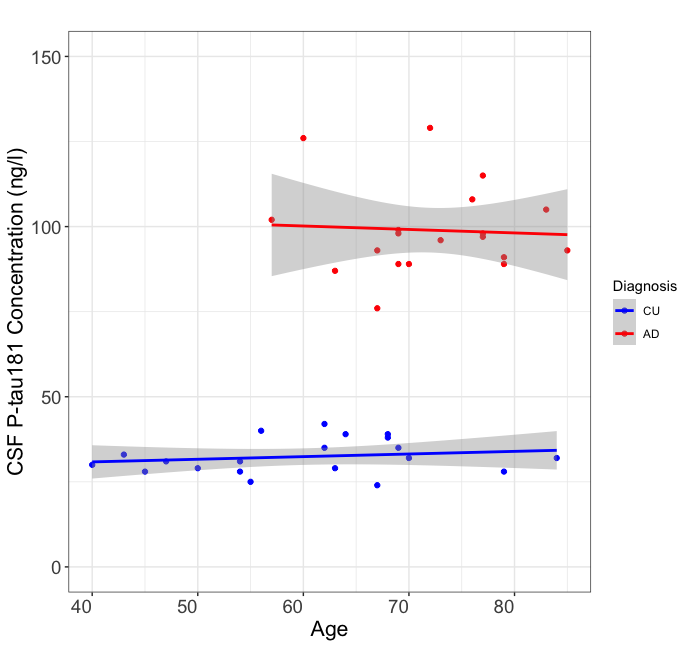

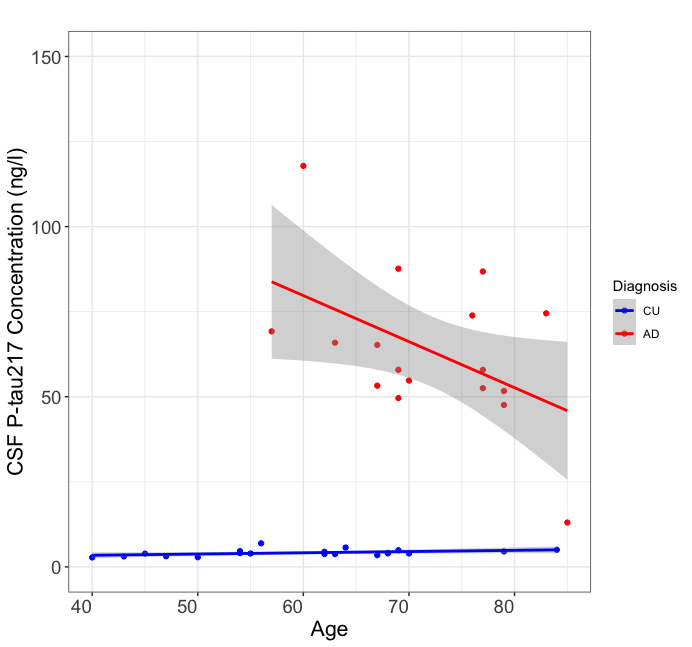

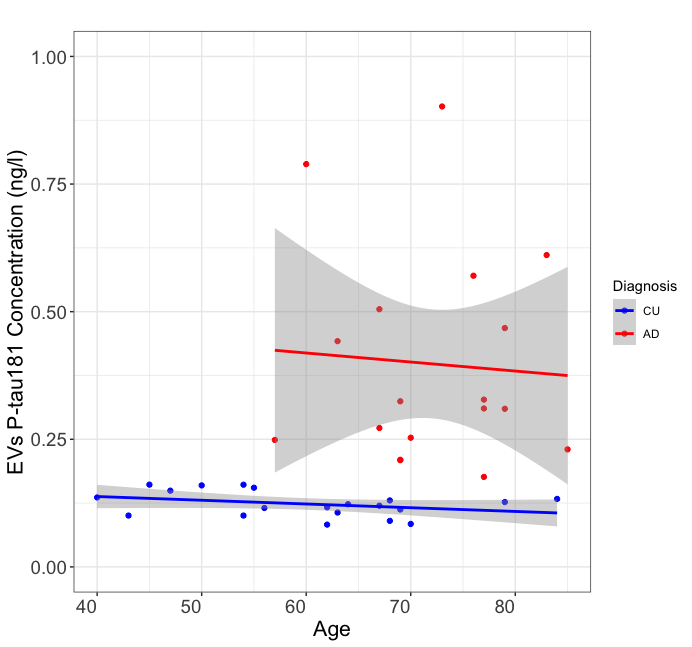

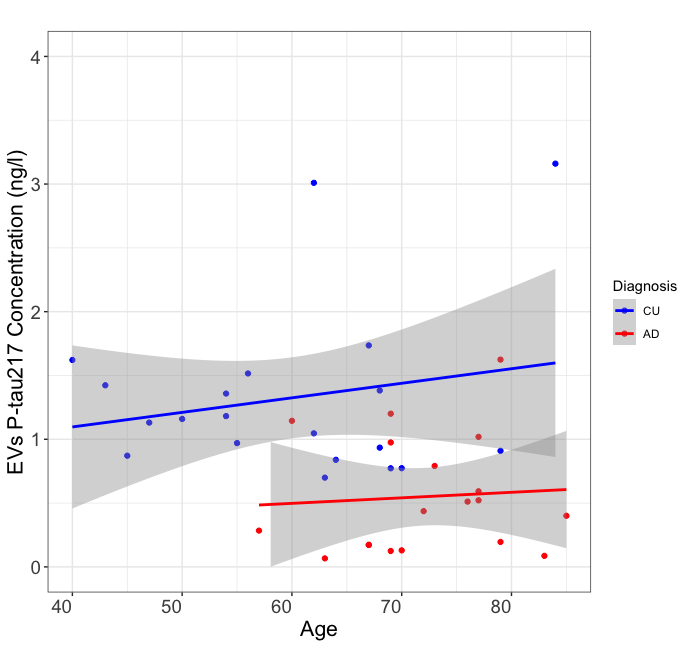


**a**

**b**

**c**

**d**

**
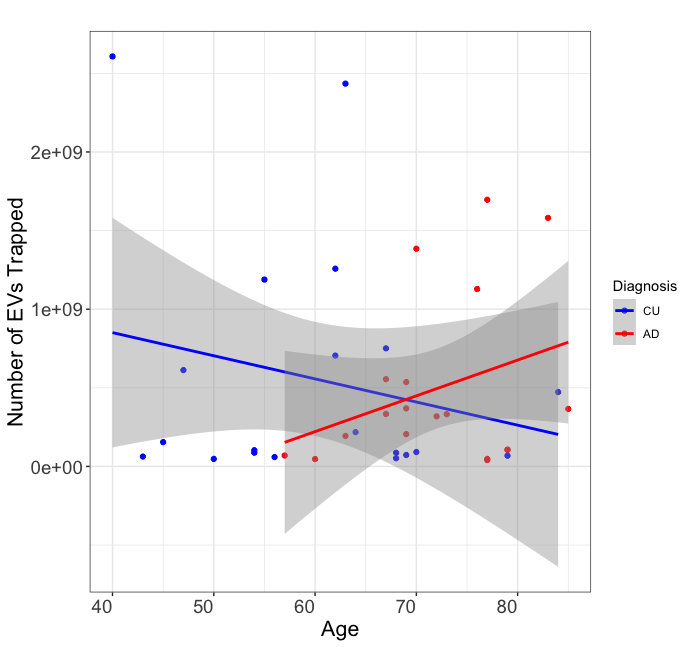
**

**Supplementary Figure 6.** **Correlation analysis between nanoparticle tracking analysis (NTA)** **-Age and EVs concentrations**. This scatter plot incorporates a linear model to illustrate the relationship between Age alongside the respective number of EVs trapped from Alzheimer’s disease (AD) patients (N=20) and cognitive unimpaired (CU) (N=20). Correlations were tested with Spearman’s rank correlation test, within the diagnostic groups. All correlations were non-significant (P>0.05).


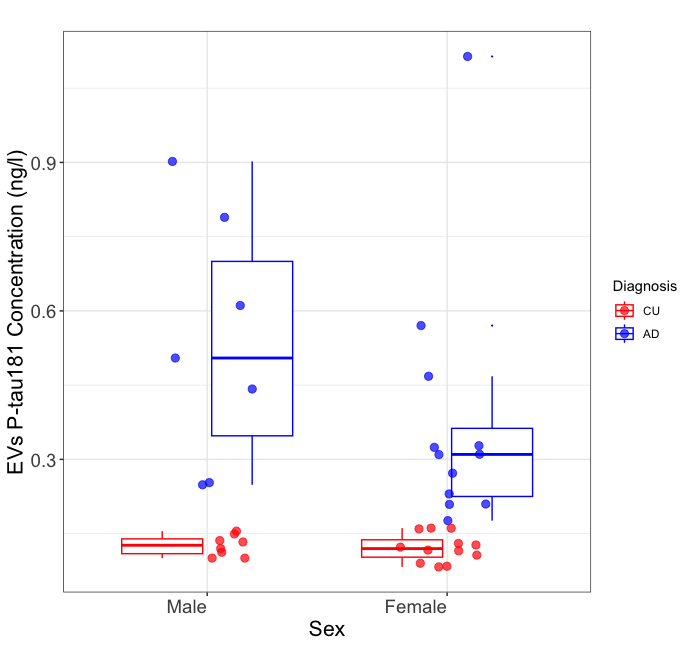

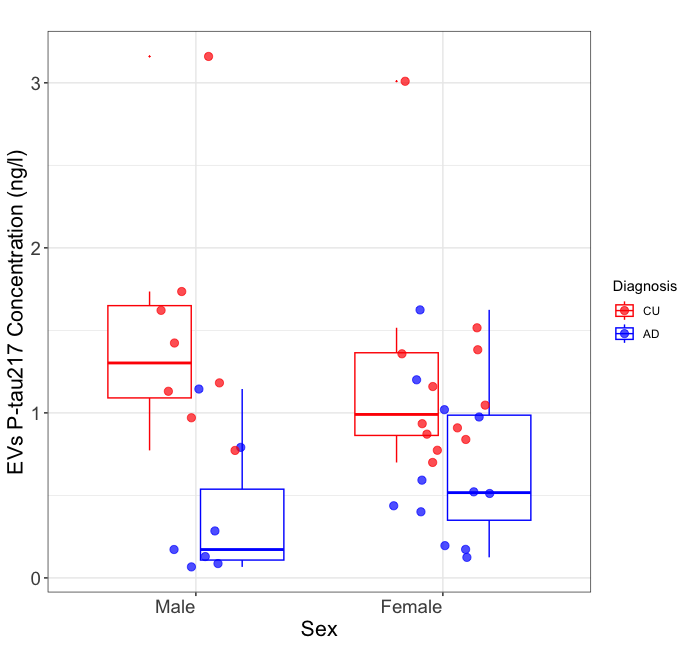


**a**

**b**

**Supplementary Figure 7. Correlation analysis between EV concentrations of P-tau217 and P-tau181 levels** **and** **Sex**. This box plot illustrate the relationship between Sex and concentrations of P-tau217 (**a**) and P-tau181(**b**) trapped from Alzheimer’s disease (AD) patients (n=20) and cognitive unimpaired (CU) (n=20). Correlations were tested with Spearman’s rank correlation test, within the diagnostic groups. All correlations were non-significant (P>0.05).
