# Supplementary material for: Phosphorylated tau in cerebrospinal fluid-derived extracellular vesicles in Alzheimer’s disease: a pilot study
Source: Sci Rep. 2024 Oct 25;14:25419. doi: 10.1038/s41598-024-75406-0 (PMC11511998; doi:10.1038/s41598-024-75406-0)
Supplement: Supplementary file 2 — Supplementary Material 2 [file 41598_2024_75406_MOESM2_ESM.docx]

**Phosphorylated Tau In Cerebrospinal Fluid-Derived Extracellular Vesicles In Alzheimer’s Disease: A Pilot Study**

*Roman* ***Sattarov****^1^****^*^****, Megan* ***Havers****^2*^, Camilla* ***Orbjörn****^1^, Erik* ***Stomrud*** *^1,3,4^, Shorena* ***Janelidze****^1,4^, Thomas* ***Laurell****^2^, Niklas* ***Mattsson-Carlgren****^1,4,5*^*

*Affiliations:*

*1 Clinical Memory Research Unit, Department of Clinical Sciences Malmö, Lund University, Lund, Sweden.*

*2 Department of Biomedical Engineering, Lund University, Lund, Sweden*

*3 Memory Clinic, Skåne University Hospital, Malmö, Sweden*

*4 Wallenberg Center for Molecular Medicine, Lund University, Lund, Sweden*

*5 Department of Neurology, Skåne University HospitalLund, Sweden*

*Roman* ***Sattarov*** *(*[Roman.Sattarov@med.lu.se](mailto:Roman.Sattarov@med.lu.se)*)*

*Megan* ***Havers*** *(Megan.Havers@bme.lth.se)*

*Camilla* ***Orbjörn*** *(*[*Camilla.Orbjorn@med.lu.se*](mailto:Camilla.Orbjorn@med.lu.se)*)*

*Erik* ***Stomrud*** *(Erik.Stomrud@med.lu.se)*

*Thomas* ***Laurell*** *(*[Thomas.Laurell@bme.lth.se](mailto:Thomas.Laurell@bme.lth.se)*)*

*Shorena* ***Janelidze*** *(*[*Shorena.Janelidze@med.lu.se*](mailto:Shorena.Janelidze@med.lu.se)*)*

*Niklas* ***Mattsson-Carlgren*** *(*[Niklas.Mattsson-Carlgren@med.lu.se](mailto:Niklas.Mattsson-Carlgren@med.lu.se)*)*

*Correspondence to R.S. ([Roman.Sattarov@med.lu.se](mailto:Roman.Sattarov@med.lu.se)), M.H. ([Megan.Havers@bme.lth.se](mailto:Megan.Havers@bme.lth.se)) and N.M.C ([Niklas.Mattsson-Carlgren@med.lu.se](mailto:Niklas.Mattsson-Carlgren@med.lu.se))

**List of abbreviations**

Alzheimer's Disease (AD)

β-Amyloid (Aβ)

Cerebrospinal Fluid (CSF)

Cognitively Unimpaired (CU)

Extracellular Vesicles (EVs)

Meso Scale Discovery (MSD)

Nanoparticle Tracking Analysis **(**NTA)

Phosphate-Buffered Saline (PBS)

Phosphorylated Tau (P-tau)

Room Temperature (RT)

Transmission Electron Microscopy **(**TEM)
